# Supplementary material for: An ancestral interaction module promotes oligomerization in divergent mitochondrial ATP synthases
Source: Nat Commun. 2022 Oct 11;13:5989. doi: 10.1038/s41467-022-33588-z (PMC9553925; doi:10.1038/s41467-022-33588-z)
Supplement: Supplementary file 1 — Supplementary Information [file 41467_2022_33588_MOESM1_ESM.pdf]

An ancestral interaction module promotes oligomerization in divergent mitochondrial ATP synthases

Ondřej Gahura[1,4], Alexander Mühleip[2,4], Carolina Hierro-Yap[1,3], Brian Panicucci[1], Minal Jain[1,3], David Hollaus[3], Martina Slapničková[1], Alena Zíková[1,3,*], Alexey Amunts[2,*]

[1]Institute of Parasitology, Biology Centre, Czech Academy of Sciences, 37005 České Budějovice, Czech Republic
[2]Science for Life Laboratory, Department of Biochemistry and Biophysics, Stockholm University, 17165 Solna, Sweden
[3]Faculty of Science, University of South Bohemia, 37005 České Budějovice, Czech Republic
[4] These authors contributed equally: Ondřej Gahura, Alexander Mühleip

[*] Correspondence to: azikova@paru.cas.cz; amunts@scilifelab.se

SUPPLEMENTARY INFORMATION

<u>Content of SI:</u>

Supplementary Figures 1-9

Supplementary Tables 1-4

Supplementary References

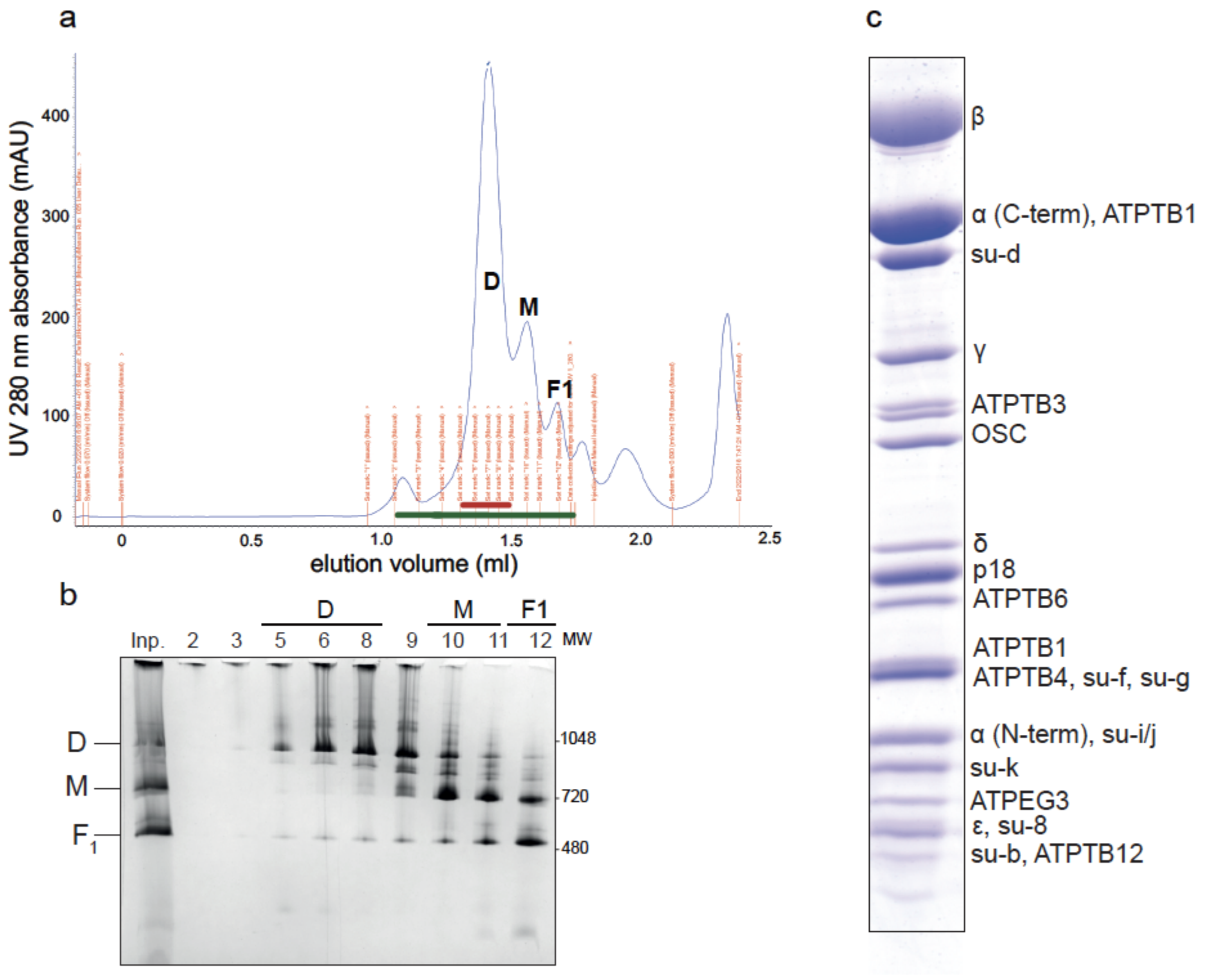

Supplementary Fig. 1. Purification of the *T. brucei* ATP synthase dimer.
a, Size exclusion chromatography trace with peaks enriched with ATP synthase dimers (D), monomers (M) and F_1-ATPase (F_1) labelled. The red bar marks the fractions for cryo-EM. **b,** Fractions from size exclusion chromatography marked with green bar in (a) resolved by native BN-PAGE. Positions of molecular weight (MW) marker are shown. **c,** Dimer-enriched fractions resolved by SDS-PAGE stained by Coomassie blue dye. Bands are annotated based on mass spectrometry identification from excised gel pieces.

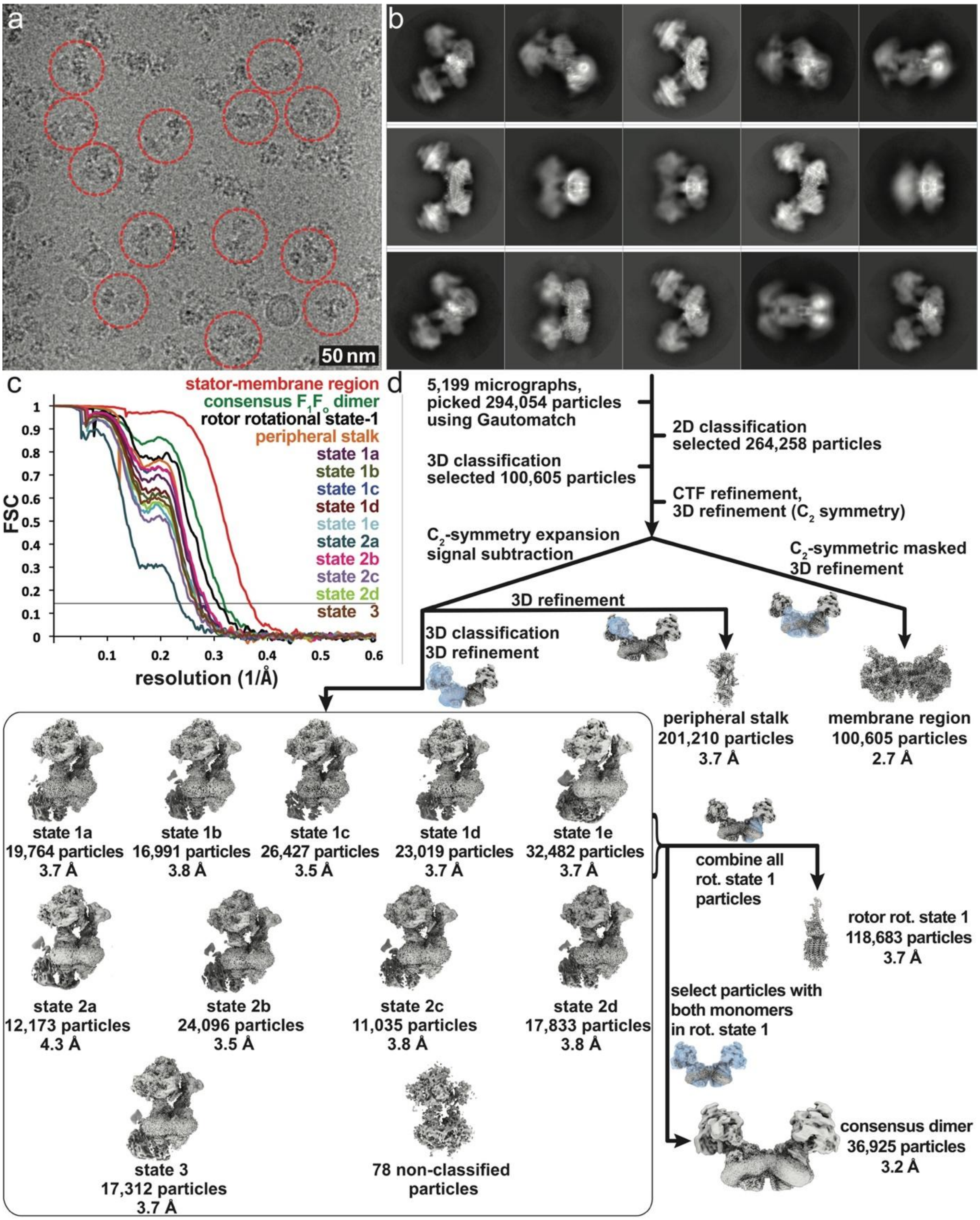

Supplementary Fig. 2. Cryo-EM data processing of the *T. brucei* ATP synthase dimer. **a**, Representative micrograph of 5199 micrographs collected in total. **b**, 2D class averages. **c**, Fourier Shell Correlation (FSC) curves showing the estimated resolutions of ATP synthase maps according to the gold standard 0.143 criterion. **d**, Data processing scheme resulting in maps covering all regions of the complex, as well as 10 rotational states.

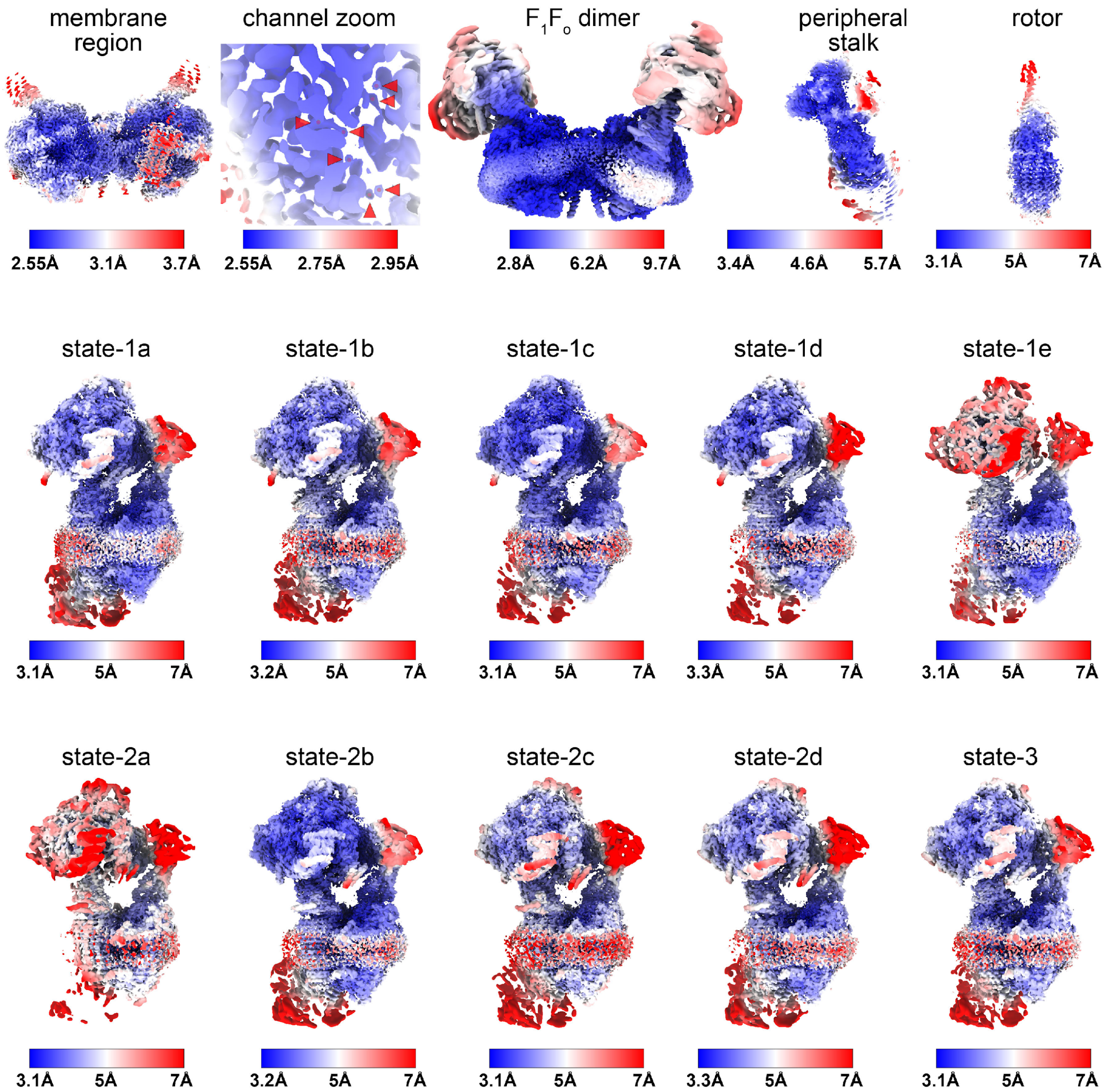

Supplementary Fig. 3. Local resolution estimation of final cryo-em maps. Local resolution estimates colored according to the respective color legends of the membrane region, F_1F_o dimer, the peripheral stalk, the rotor and all identified rotational states. A zoomed-in view of the membrane region shows that the resolution in the lumenal channel extends to 2.55 Å, allowing the assignment of water molecules.

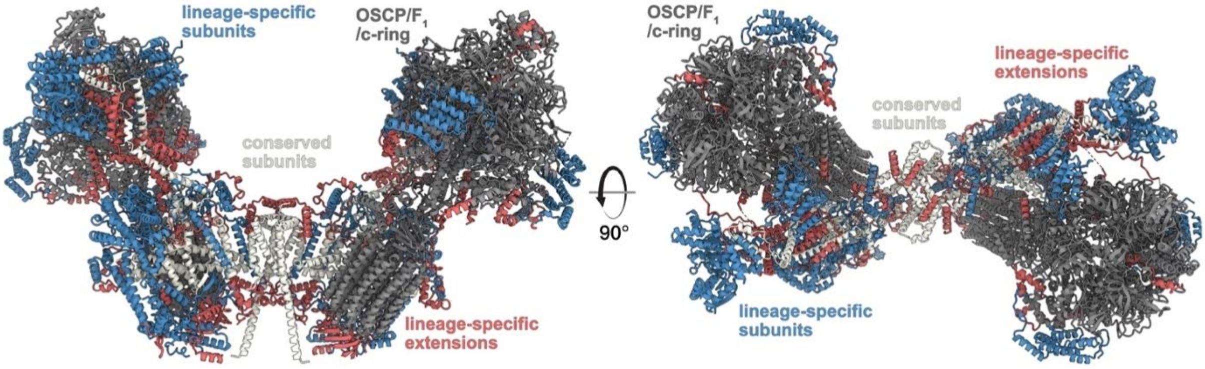

Supplementary Fig. 4. Conserved and phylum specific elements generate the *T. brucei* ATP synthase architecture.

The canonical OSCP/F_1/*c*-ring monomers (dark grey) are tied together by both conserved F_o subunits and extensions of lineage-specific subunits (red). The F_o periphery and peripheral stalk attachment are composed of lineage specific subunits (blue).

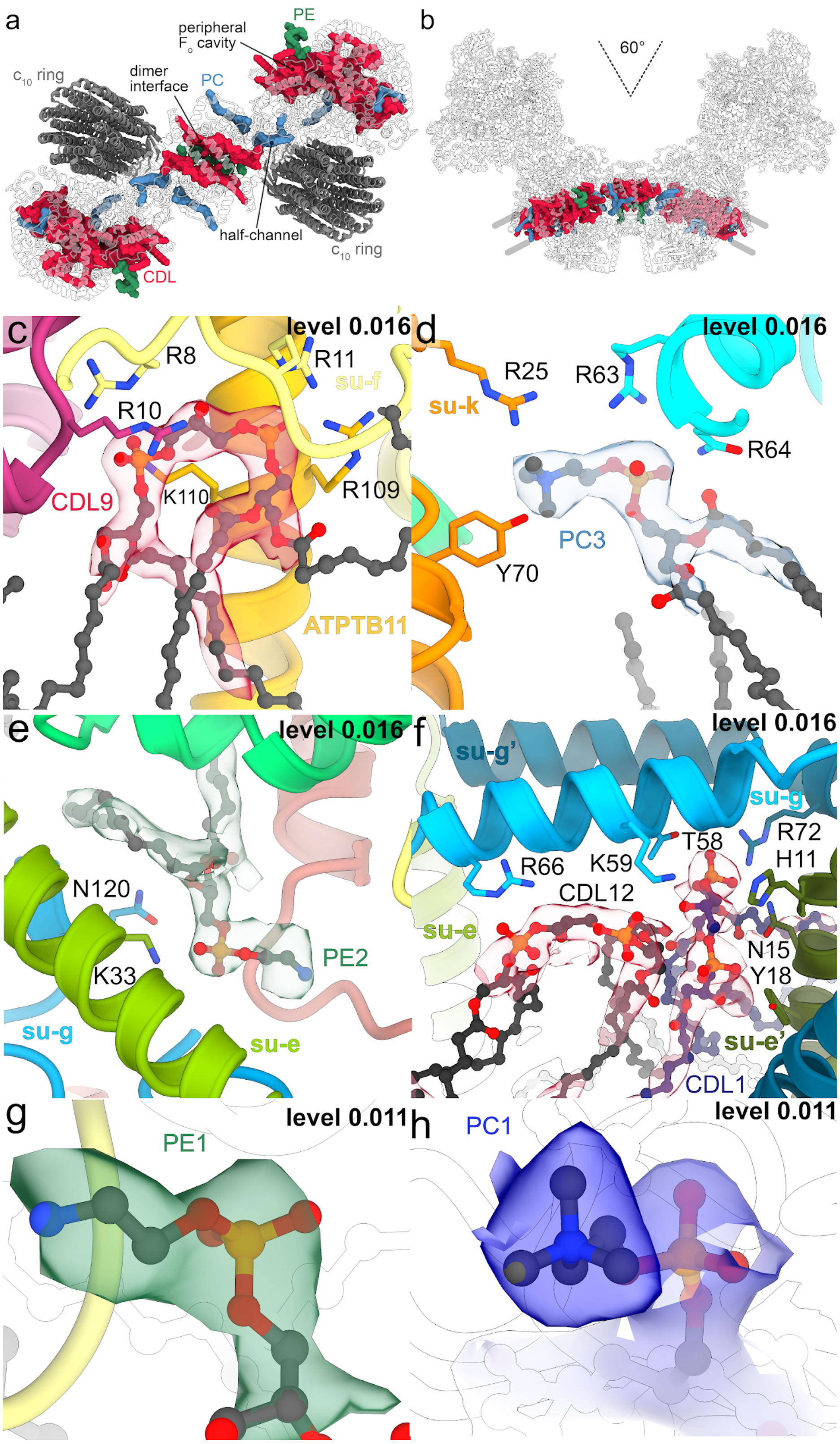

Supplementary Fig. 5. The F_o region coordinates numerous bound lipids.
a, F_o top view, cardiolipin (CDL), phosphatidylcholine (PC) and phosphatidlyethanolamine (PE) are bound at the dimer interface, the lumenal proton half-channel and the peripheral F_o cavity. **b**, The 60°-dimer angle generates a curved F_o region with phospholipids bound in an

arc-shaped bilayer. **c-f**, Bound lipids with cryo-EM density and coordinating residues. **g-h,** Representative densities of headgroups of PE (g) and PC (h).

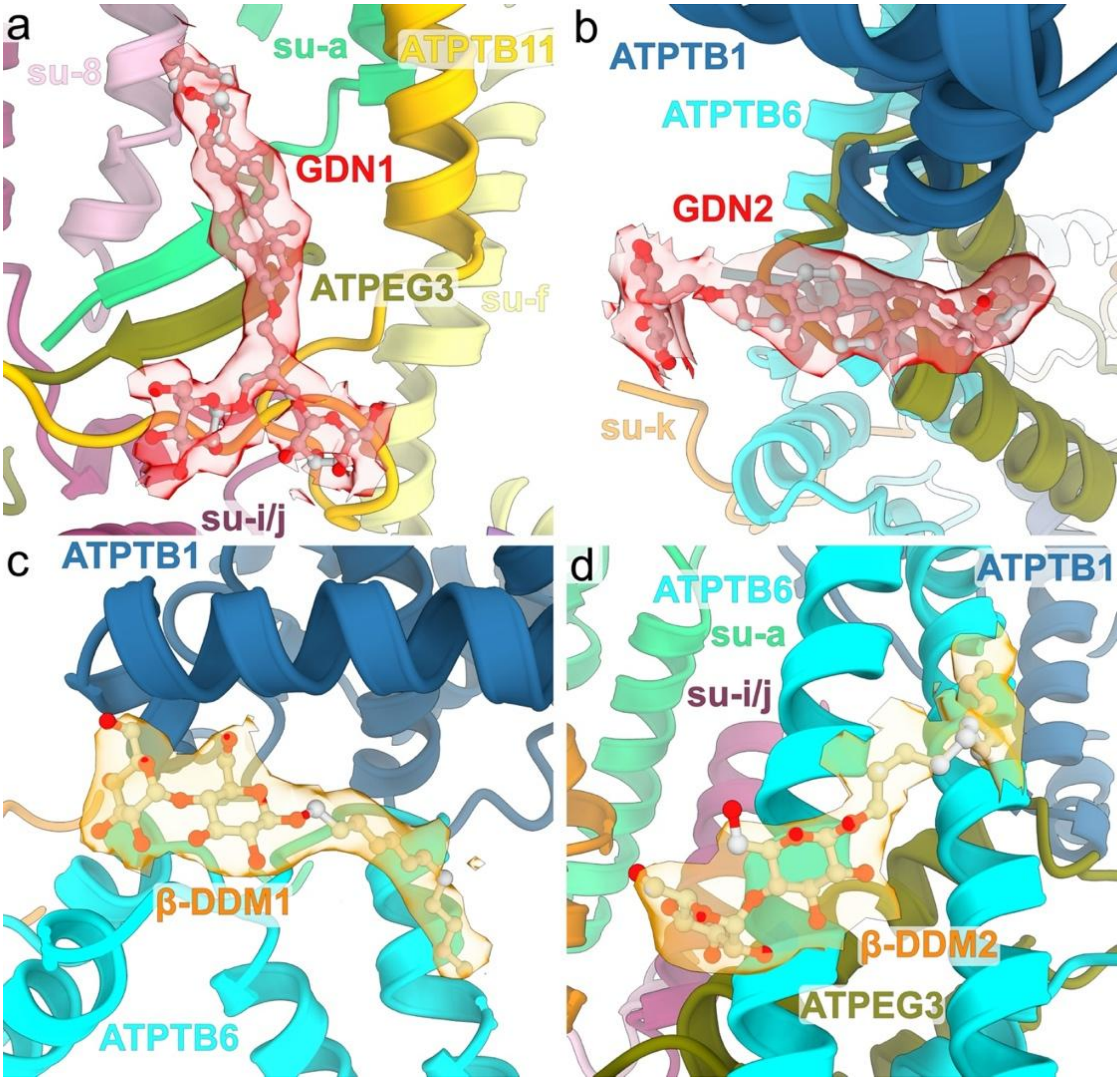

Supplementary Fig. 6. Bound detergents of the F_o region.
GDN **(a,b)** and β-DDM **(c,d)** molecules bound in the periphery of the membrane region with cryo-EM map densities shown (transparent), indicating that both glycosides are retained in the detergent micelle.

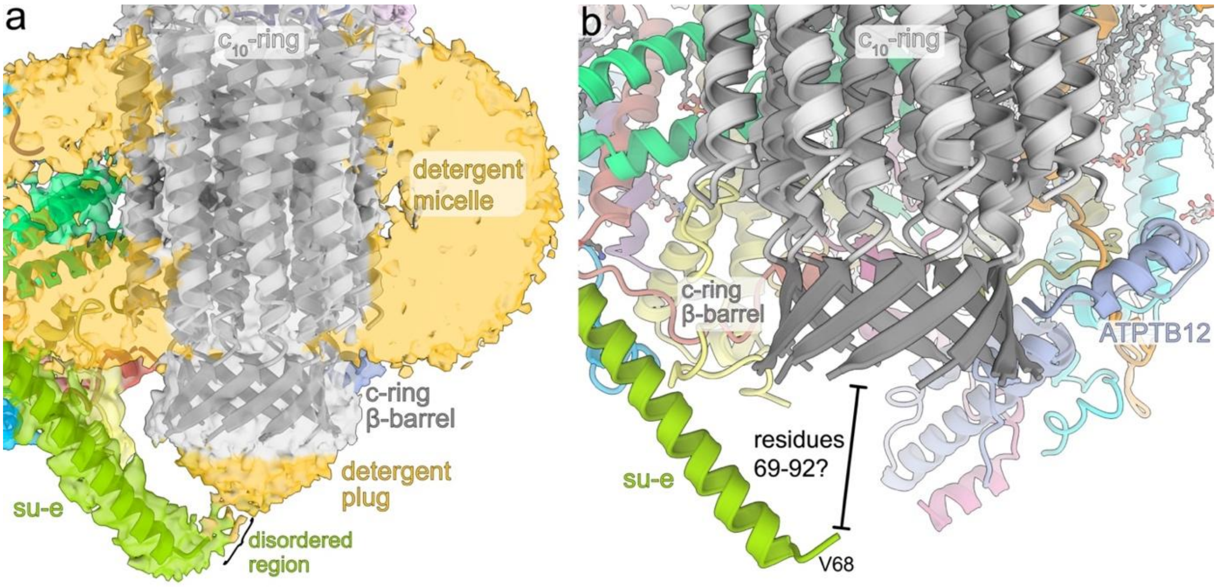

Supplementary Fig. 7. The C-terminal tail of subunit-*e* interacts with the c_{10}-ring.
a, The cryo-EM map reveals disordered detergent density of the detergent belt surrounding the membrane region as well as a detergent plug on the luminal side of the *c*-ring. **b,** The helical C-terminus of subunit-*e* extends into the lumen towards the *c*-ring. The terminal 23 residues are disordered and likely interact with the ®-barrel.

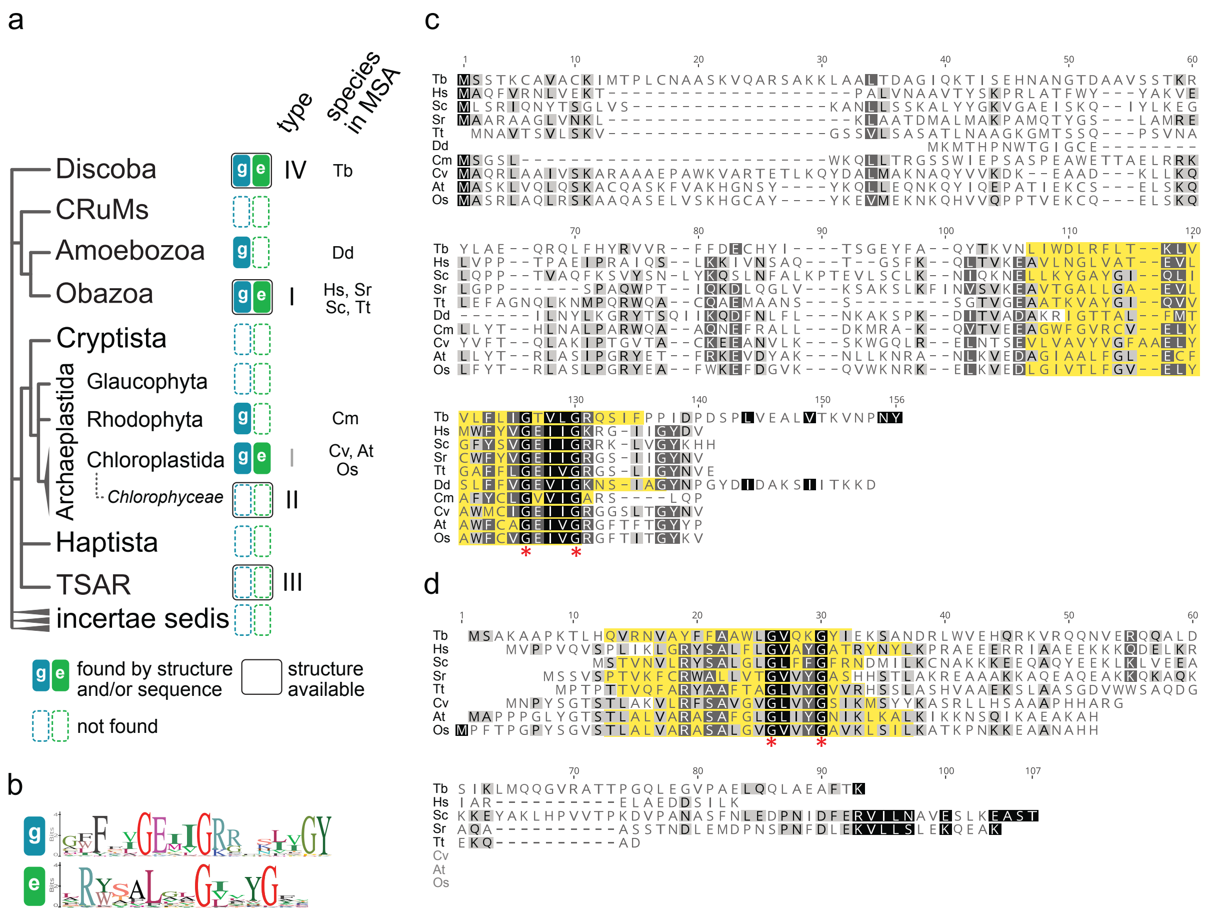

Supplementary Fig. 8. Phylogenetic distribution and sequence conservancy of subunit-*e* and -*g*.

a, Distribution of subunits *e* and *g* mapped on the phylogenetic tree of eukaryotes[3]. Homologs of subunits *e* and *g* were searched in non-redundant GenBank and UniprotKB protein databases by PSI-BLAST, and phmmer and hmmsearch[4], respectively, using individual sequences of representatives from *H. sapiens* and *T. brucei*, and in the case of hmmsearch a multiple sequence alignment (MSA) of representatives from *Homo sapiens*, *Saccharomyces cerevisiae*, *Arabidopsis thaliana* and *T. brucei*, as queries. Groups, in which at least one structure of ATP synthase is available, are marked. Abbreviations of species used in MSA in panels (c) and (d) are shown. **b**, Sequence logo of GXXXG motifs and flanking regions of subunits *e* and *g*. Hits from hmmsearch were clustered by CD-HIT Suite[5] to 50% sequence identity and MSA of representative sequences of each cluster was generated by Clustal Omega4[6]. The sequence logos were created from MSA in Geneious Prime (Biomatters Ltd.). **c**,**d**, MSA of sequences of subunits g (c) and e (d) from species representing major groups shown in (a) generated by MUSCLE[7] and visualized in Geneious Prime. The experimentally determined or predicted transmembrane regions are highlighted in yellow. Species abbreviations: Tb – *T. brucei*, Hs – *H. sapiens*, Sc – *S. cerevisiae*, Sr – *Salpingoeca rosetta*, Tt – *Thecamonas trahens*, Dd – *Dictyostelium discoideum*, Cm – *Cyanidioschyzon merolae*, Cv – *Chlorella vulgaris*, At – *Arabidopsis thaliana*, Os – *Oryza sativa*.

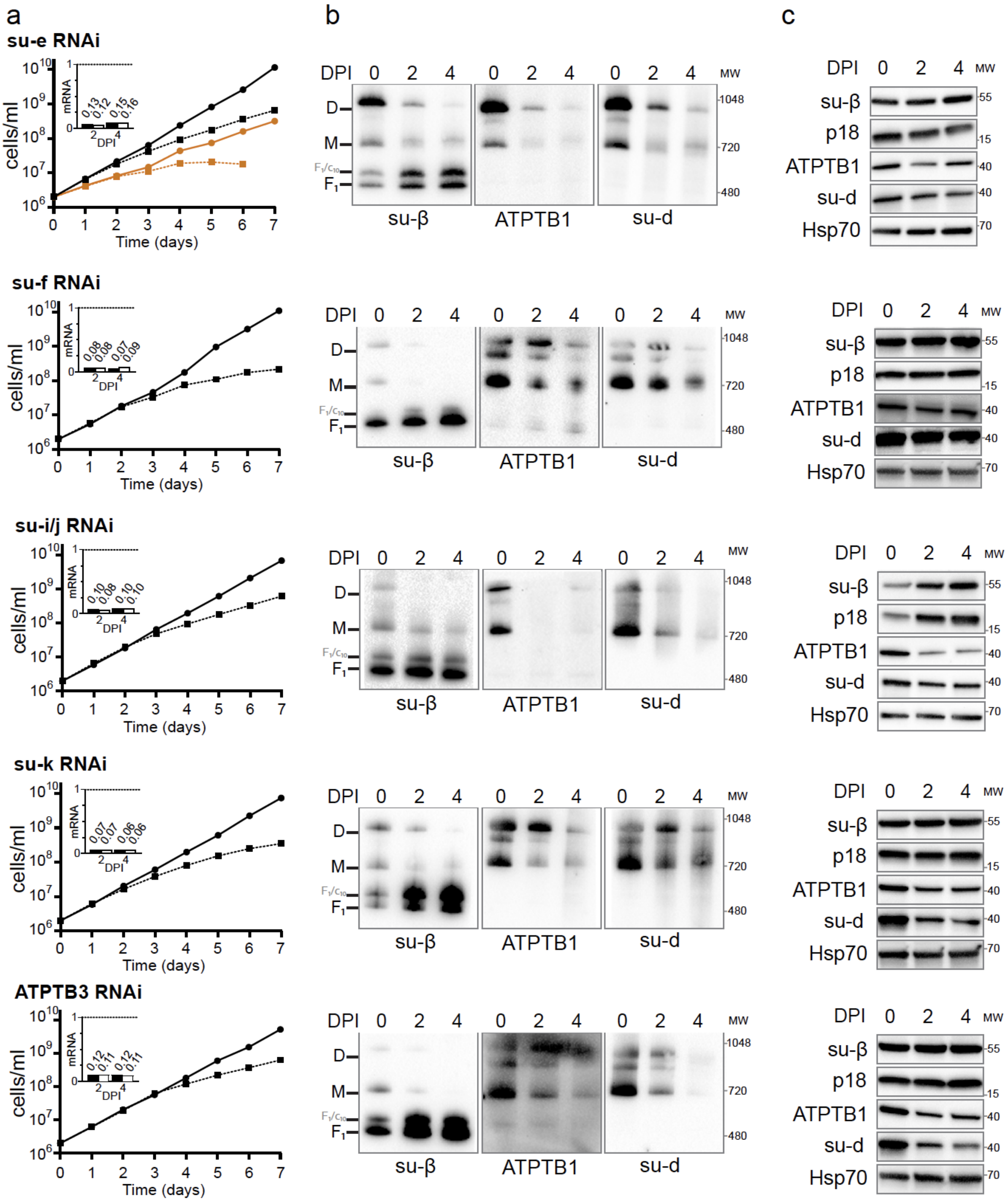
a
b
c
su-e RNAi
su-f RNAi
su-i/j RNAi
su-k RNAi
ATPTB3 RNAi
cells/ml
Time (days)
mRNA
DPI
0.13 0.12 0.15 0.16
0.08 0.08 0.07 0.09
0.10 0.08 0.10 0.10
0.07 0.07 0.06 0.06
0.12 0.11 0.12 0.11
DPI 0 2 4
MW
D
M
F1/c10
F1
1048
720
480
su-β
ATPTB1
su-d
p18
Hsp70
55
15
40
70

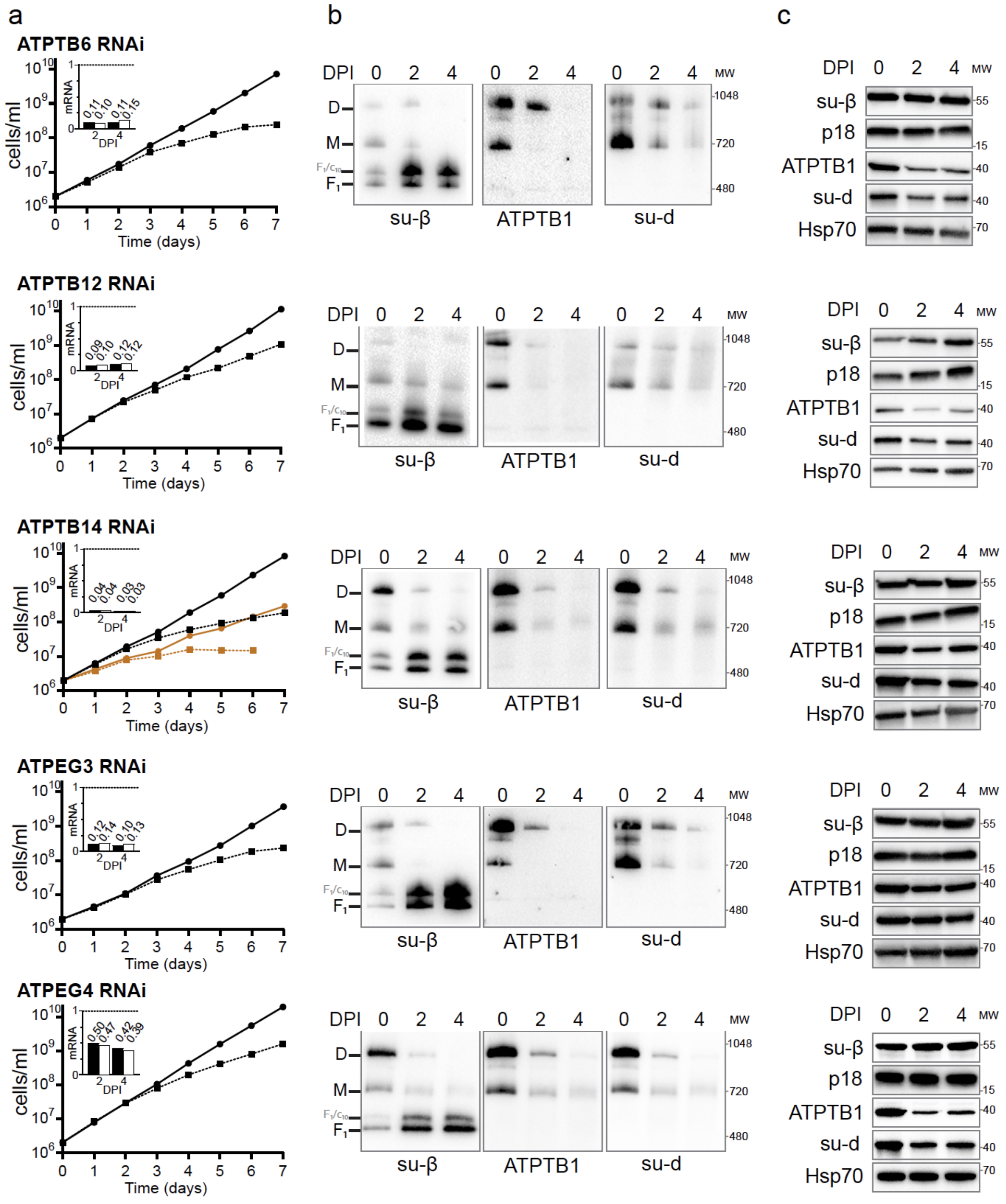

Supplementary Fig. 9. Effects of RNAi knock-down of ATP synthase subunits on viability and stability and dimerization of ATP synthase.

a, Growth curves of indicated non-induced (solid lines) and tetracycline induced (dashed lines) RNAi cells lines in the presence (black) or absence (brown) of glucose. The insets show relative levels of the respective target mRNA at indicated days post induction (DPI) normalized to the levels of 18S rRNA (black bars) or ß-tubulin (white bars). **b**, Immunoblots of mitochondrial lysates from indicated RNAi cell lines resolved by BN-PAGE probed by antibodies against indicated ATP synthase subunits (n=1). Positions of molecular weight (MW) marker are shown. **c**, Immunoblots of whole cell lysates from indicated RNAi cell lines probed with indicated antibodies (n=1). Positions of MW marker are shown.

	Membrane region	Rotor	Peripheral stalk	F_1F_o dimer	Rot. 1a	Rot. 1b	Rot. 1c	Rot. 1d	Rot. 1e	Rot. 2a	Rot. 2b	Rot. 2c	Rot. 2d	Rot. 3
**Data collection**														
**Microscope**	Titan Krios													
**Voltage (kV)**	300													
**Camera**	K2 Summit													
**Magnification**	165 kx													
**Exposure (e^-/Å^2)**	33													
**Defocus range (µm)**	-1.6 to -3.2													
**Pixel size (Å)**	0.83													
**Movies collected**	5,199													
**Frames per movie**	20													
**Data processing**														
**Initial particles**	100,605 (C_2 symmetry-expanded: 201,210)													
**Final no. particles**	100,605	118,683	201,210	36,925	19,764	26,427	23,019	16,991	34,482	12,173	24,096	11,035	17,833	17.312
**Symmetry**	C_2	C_1	C_1	C_2	C_1	C_1	C_1	C_1	C_1	C_1	C_1	C_1	C_1	C_1
**Map resolution (Å)**	2.7	3.7	3.7	3.2	3.7	3.5	3.7	3.8	3.7	4.3	3.5	3.8	3.8	3.7
**Sharpening B factor**	-46.2	-74.4	-92.5	-49.8	-61.8	-61.1	-57.6	-45.6	-58.0	-73.8	-54.5	-65.2	-54.9	-61.7
**EMD ID**														
**Model refinement statistics**														
**CC (map/model)**	0.86	0.83	0.82	0.71	0.79	0.79	0.82	0.79	0.69	0.71	0.81	0.77	0.77	0.79
**Resolution (map/model)**	2.65	3.4	3.68	3.13	3.48	3.56	3.36	3.55	3.57	3.94	3.39	3.73	3.64	3.58
**No. of atoms**	76,690	19,669	12,083	251,552	129,568	129,568	129,568	129,568	129,568	129,563	129,563	129,563	129,563	129,566
**No. of residues**	4074	1285	767	15,356	7872	7872	7872	7872	7872	7872	7872	7872	7872	7872
**No. of lipids**	36	0	0	36	21	21	21	21	21	21	21	21	21	21
**No. of ATP/ADP**	0	0	0	10	5	5	5	5	5	5	5	5	5	5
**No. of Mg ions**	0	0	0	10	5	5	5	5	5	5	5	5	5	5
**B-factor (Å^2)**														
**- protein**	54.05	56.13	77.88	84.48	55.65	70.37	80.22	83.27	70.70	112,72	79.93	65.52	66.49	101.5
**- ligands**	50.57	58.25	-	69.94	40.99	72.29	63.18	78.43	63.76	75,25	74.47	61.79	46.55	83.68
**Rotamer outliers (%)**	0.44	0.40	0.31	0.22	0.42	0.09	0.18	0.26	0.58	0.18	0.27	0.48	0.42	0.39
**Ramachandran (%)**														
**- outliers**	0.00	0.00	0.00	0.01	0.001	0.003	0.004	0.01	0.003	0.01	0.00	0.04	0.04	0.04
**- allowed**	1.57	1.91	1.59	1.56	1.52	1.65	1.44	1.49	1.49	1.67	1.58	1.47	1.65	1.79
**- favored**	98.43	98.08	98.41	98.42	98.47	98.34	98.56	98.49	98.48	98.31	98.42	98.49	98.31	98.17
**Clash score**	1.66	2.44	2.32	2.26	2.60	2.65	2.53	2.67	2.99	2.38	2.30	2.52	2.38	3.57
**MolProbity score**	0.92	1.03	1.01	1.00	1.05	1.05	1.04	1.05	1.09	1.02	1.01	1.04	1.02	1.15
**RMSD**														
**- bonds (Å)**	0.004	0.004	0.02	0.003	0.003	0.003	0.004	0.003	0.003	0.002	0.003	0.003	0.003	0.003
**- angles (°)**	0.455	0.416	0.386	0.407	0.414	0.424	0.417	0.407	0.412	0.410	0.416	0.419	0.428	0.421
**EMRinger score**	5.11	3.96	1.61	2.56	3.24	2.95	3.32	2.85	3.32	1.35	2.89	2.32	2.49	2.8
**PDB ID**														

Supplementary Table 1. Data collection, processing, model refinement and validation statistics.

Subunit name	TriTrypDB Lister strain 427 ID	TriTrypDB TREU927 strain ID	Uniprot TREU927 strain ID	Residues	Residues built
**F_1 subcomplex**					
〈	Tb427_070081800<br>Tb427_070081900	Tb927.7.7420<br>Tb927.7.7430	Q57TX9	584	45-151,<br>161-584
®	Tb427_030013500	Tb927.3.1380	Q57XX1	519	26-514
©	Tb427_100005200	Tb927.10.180	B0Z0F6	305	2-301
™	Tb427_060054900	Tb927.6.4990	Q586H1	182	22-182
∑	Tb427_100054600	Tb427.10.5050	N/A	75	11-75
**p18**	Tb427_050022900	Tb927.5.1710	Q57ZP0	188	23-188
**F_o subcomplex**					
**OSCP**	Tb427_100087100	Tb927.10.8030	Q38AG1	255	18-202,<br>208-255
a	mt encoded	mt encoded	P24499	231	1-231
b	Tb427_040009100	Tb927.4.720	Q580A0	105	26-105
c	Tb427_100018700<br>Tb427_110057900<br>Tb427_070019000	Tb927.10.1570<br>Tb927.11.5280<br>Tb927.7.1470	Q38C84<br>Q385P0<br>Q57WQ3	118	41-118
d	Tb427_050035800	Tb927.5.2930	Q57ZW9	370	17-325,<br>332-354
e	Tb427_110010200	Tb927.11.600	N/A	92	1-383
f	Tb427_030016600	Tb927.3.1690	Q57ZE2	145	2-136
g	Tb427_020016900	Tb927.2.3610	Q586X8	144	16-144
i/j	Tb427_030029400	Tb927.3.2880	Q57ZM4	104	2-104
k	Tb427_070011800	Tb927.7.840	Q57VT0	124	20-124
**8**	Tb427_040037300	Tb927.4.3450	Q585K5	114	29-114
**ATBTB1**	Tb427_100008400	Tb927.10.520	Q38CI8	396	1-383
**ATPTB3**	Tb427_110067400	Tb927.11.6250	Q385E4	269	2-269
**ATPTB4**	Tb427_100105100	Tb927.10.9830	Q389Z3	157	21-157
**ATPTB6**	Tb427_110017200	Tb927.11.1270	Q387C5	169	2-169
**ATPTB11**	Tb427_030021500	Tb927.3.2180	Q582T1	156	18-156
**ATPTB12**	Tb427_050037400	Tb927.5.3090	Q57Z84	101	5-100
**ATPEG3**	Tb427_060009300	Tb927.6.590	Q583U4	98	14-98
**ATPEG4**	N/A	Tb927.11.2245	N/A	62	1-62

Supplementary Table 2. Composition of *T. brucei* ATP synthase dimer.

Subunit	Primer pair sequences
**Primers for amplification of RNAi cassettes**	
b	TAATCTCGAGGGTACCGTTGAGTGAGGAGGAACGGG<br>GCAGTCTAGAGGATCCTATCCCTTCCACCCACCACT
e	TAATCTCGAGGGTACCGGGAGTACAGAAGGGCTACA<br>TAGATCTAGAGGATCCCGTGCACACCATCAGCTG
f	ATACTCGAGGGTACCGTGAGTACCGCCTTTACGC<br>GCGTCTAGAGGATCCAGCACTGATCACCAAACTGC
g	ACTGCTCGAGGGTACCACGCGGGAATTCAAAAGACC<br>GCGGTCTAGAGGATCCCGTTGCGGTGCTTGTCATTA
i/j	TAATCTCGAGGGTACCGAATATCCGATGCATGCCGC<br>GCCGTCTAGAGGATCCACTTCGCTCTACTGCATGCA
k	ATTACTCGAGCCCGGGCGATCAGTGCAGGGGATTTT<br>GCCGTCTAGAGGATCCTTTCCTCGAAAACGCACACA
**8**	ATGACTCGAGGGTACCGGGCTATGGTGTGGTATTATGC<br>GACGTCTAGAGGATCCGCAGAAAACTCCCAACGACA
**ATPTB3**	ACTGCTCGAGGGTACCAAAGAGGAGGTGAGGTCTGC<br>GCAGTCTAGAGGATCCCCCTAGGGTTCTTCGAAGCA
**ATPTB4**	CTGACTCGAGGGTACCTTCCTTTTCTGCTGCATCGG<br>GCAGTCTAGAGGATCCCTCCTCGGGCTTCCAATTTG
**ATPTB6**	ACTGCTCGAGGGTACCCAACATGGCAGTATCCGGTG<br>GCAGTCTAGAGGATCCTTATTAGTGGCGGTGGTGGT
**ATPTB11**	ACTGCTCGAGGGTACCGCGCTCGTCTTCTCCATTTC<br>GCAGAAGCTTGGATCCAGGTTGGGGTGTTTAGGGAG
**ATPTB12**	TAATCTCGAGGGTACCGACGCCATCAAAGGAATGCC<br>GCCGTCTAGAGGATCCAGCAGCCAACAAACAGACAA
**ATPEG3**	TACACTCGAGGGTACCAAACCTGAAGGCCCTCACAC<br>GCAGTCTAGAGGATCCCTCTTTCGTGCCGCCTGATA
**Primers for quantification of mRNA levels by qPCR**	
b	CCAAGAGTGATGATGGCCCC<br>CGTTTAGGGTCGCGGAAAAC
e	CAAGCCTTGCACACACTTTATG<br>CCGCAAAGAAGTACGCCAC
f	TTTTCTACATACCGCAGCAGT<br>TACCATTCCATGCGCGTTG
g	GCAATTGTGTGAGCTGAACG<br>TACTGGCCGCATTGCATAAC
i/j	AGAGTAAAAGCGCGCCTACG

	CAGTTGGAAAACCGGTAGCC
k	ACACAAAACACTTCCAGCAGA<br>CGCTATGACGGACAGGTGT
**8**	GCTACGGCGACTTGGTGC<br>CGTCACCGCGTATTTGTTCA
**ATPTB3**	AACGTTTATATCAGCGGGCG<br>CTGTTTTGGTCTGCACACGA
**ATPTB4**	CCAAACTTTGAAGCAGCGGA<br>ATTCCTTGGATCCGCACCTT
**ATPTB6**	TCGGCATAGGAGAAGTAACGA<br>GATTCGGTTTGGAACTTGCG
**ATPTB11**	CAACGGCCCCACATTCTC<br>ACACCGCGGTCATTCATTG
**ATPTB12**	GCACTTCATTCTCCCGACTG<br>ACATGATGTAACACCTCCGC
**ATPEG3**	TGGCCCCACATGACTGAAAA<br>GGAAGTGATCCGCCGGATTT

Supplementary Table 3. List of primers used in the study.

Target	Type	Reference	Dilution SDS-PAGE	Dilution BN-PAGE
**Primary antibodies**				
**subunit-β**	rabbit polyclonal	8	1:2000	1:2000
**p18**	rabbit polyclonal	8	1:1000	-
**ATPTB1**	rabbit polyclonal	8	1:1000	1:1000
**subunit-*d***	rabbit polyclonal	8	1:1000	1:500
**mtHsp70**	mouse monoclonal	9	1:5000	-
**Secondary antibodies**				
**goat anti-rabbit IgG HRP conjugate**		BioRad 1721019	1:2000	1:2000
**goat anti-mouse IgG HRP conjugate**		BioRad 1721011	1:2000	1:2000

Supplementary Table 4. List of antibodies used in the study.

Supplementary References:

1. Muhleip, A., McComas, S.E. & Amunts, A. Structure of a mitochondrial ATP synthase with bound native cardiolipin. *Elife* **8**, e51179 (2019).
2. Larkin, M.A. et al. (2007). Clustal W and Clustal X version 2.0. *Bioinformatics*, **23**, 2947-2948 (2007).
3. Burki, F., Roger, A.J., Brown, M.W. & Simpson, A.G.B. The New Tree of Eukaryotes. *Trends Ecol Evol* **35**, 43-55 (2020).
4. Protein Sequence Similarity Search. *Curr Protoc Bioinformatics* **60**, 3151-31523 (2017).
5. Huang, Y., Niu, B., Gao, Y., Fu, L. & Li, W. CD-HIT Suite: a web server for clustering and comparing biological sequences. *Bioinformatics* **26**, 680-2 (2010).
6. Sievers, F. et al. Fast, scalable generation of high-quality protein multiple sequence alignments using Clustal Omega. *Mol Syst Biol* **7**, 539 (2011).
7. Edgar, R.C. MUSCLE: multiple sequence alignment with high accuracy and high throughput. *Nucleic Acids Res* **32**, 1792-7 (2004).
8. Subrtova, K., Panicucci, B. & Zikova, A. ATPaseTb2, a unique membrane-bound F_oF_1-ATPase component, is essential in bloodstream and dyskinetoplastic trypanosomes. *PLoS Pathog* **11**, e1004660 (2015).
9. Panigrahi, A.K. et al. Mitochondrial complexes in Trypanosoma brucei: a novel complex and a unique oxidoreductase complex. *Mol Cell Proteomics* **7**, 534-45 (2008).
