# Supplementary material for: An ancestral interaction module promotes oligomerization in divergent mitochondrial ATP synthases
Source: Nat Commun. 2022 Oct 11;13:5989. doi: 10.1038/s41467-022-33588-z (PMC9553925; doi:10.1038/s41467-022-33588-z)
Supplement: Supplementary file 3 — Description of Additional Supplementary Files [file 41467_2022_33588_MOESM3_ESM.pdf]

Description of Additional Supplementary Files

File Name: Supplementary Movie 1
Description: Overall structure and subunits -*g* and -*e*.

File Name: Supplementary Movie 2
Description: Rotary states 1a and 1b show tilting motion.

File Name: Supplementary Movie 3
Description: Bending of the apical accommodates the F_1/c_{10}-ring subcomplex.
