# Supplementary material for: An ancestral interaction module promotes oligomerization in divergent mitochondrial ATP synthases
Source: Nat Commun. 2022 Oct 11;13:5989. doi: 10.1038/s41467-022-33588-z (PMC9553925; doi:10.1038/s41467-022-33588-z)
Supplement: Supplementary file 7 — Reporting Summary [file 41467_2022_33588_MOESM7_ESM.pdf]

nature research

Corresponding author(s): A. Amunts

Last updated by author(s): Sep 7, 2022

Reporting Summary

Nature Research wishes to improve the reproducibility of the work that we publish. This form provides structure for consistency and transparency in reporting. For further information on Nature Research policies, see our Editorial Policies and the Editorial Policy Checklist.

Statistics

For all statistical analyses, confirm that the following items are present in the figure legend, table legend, main text, or Methods section.

n/a	Confirmed	
☐	☒	The exact sample size (*n*) for each experimental group/condition, given as a discrete number and unit of measurement
☐	☒	A statement on whether measurements were taken from distinct samples or whether the same sample was measured repeatedly
☐	☒	The statistical test(s) used AND whether they are one- or two-sided<br>*Only common tests should be described solely by name; describe more complex techniques in the Methods section.*
☒	☐	A description of all covariates tested
☒	☐	A description of any assumptions or corrections, such as tests of normality and adjustment for multiple comparisons
☐	☒	A full description of the statistical parameters including central tendency (e.g. means) or other basic estimates (e.g. regression coefficient) AND variation (e.g. standard deviation) or associated estimates of uncertainty (e.g. confidence intervals)
☐	☒	For null hypothesis testing, the test statistic (e.g. *F*, *t*, *r*) with confidence intervals, effect sizes, degrees of freedom and *P* value noted<br>*Give P values as exact values whenever suitable.*
☒	☐	For Bayesian analysis, information on the choice of priors and Markov chain Monte Carlo settings
☒	☐	For hierarchical and complex designs, identification of the appropriate level for tests and full reporting of outcomes
☒	☐	Estimates of effect sizes (e.g. Cohen's *d*, Pearson's *r*), indicating how they were calculated

Our web collection on statistics for biologists contains articles on many of the points above.

Software and code

Policy information about availability of computer code

Data collection	The datasets were collected EPU 1.9 software on FEI Titan Krios (FEI/Thermofischer) transmission electron microscope operated at 300 keV with a slit width of 20 eV on a GIF quantum energy filter (Gatan). A K2 Summit detector (Gatan) was used at a pixel size of 0.83 Å (magnification of 165,000x) with an exposure rate of 33 electrons/pixel/second fractionated over 20 frames. A defocus range of -1.6 to -3.2 µm was used.
Data analysis	Movie frames were aligned and averaged by global and local motion corrections by MotionCor2. Contrast transfer function (CTF) parameters were estimated by GCTF. Particles were picked and classified by RELION 3.0 within the Scipion 2 framework. The models were built with Bucaneer and Coot 0.9.5 and stereochemical refinement was performed using phenix.real_space_refine in the PHENIX 1.17.1 suite. Model statistics were generated using MolProbity and EMRinger. The final figures were generated using Chimera X 0.91. The proton half-channels analysis, HOLLOW was used.

For manuscripts utilizing custom algorithms or software that are central to the research but not yet described in published literature, software must be made available to editors and reviewers. We strongly encourage code deposition in a community repository (e.g. GitHub). See the Nature Research guidelines for submitting code & software for further information.

Data

Policy information about availability of data

All manuscripts must include a data availability statement. This statement should provide the following information, where applicable:

- Accession codes, unique identifiers, or web links for publicly available datasets
- A list of figures that have associated raw data
- A description of any restrictions on data availability

The atomic coordinates generated in this study have been deposited in the Protein Data Bank (PDB) under the accession codes: 8AP7 [https://www.rcsb.org/

structure/8AP7] (membrane-region), 8AP8 [https://www.rcsb.org/structure/8AP8] (peripheral stalk), 8AP9 [https://www.rcsb.org/structure/8AP9] (rotor), 8AP6 [https://www.rcsb.org/structure/8AP6] (F1Fo dimer), 8APA [https://www.rcsb.org/structure/8APA] (rotational state 1a), 8APB [https://www.rcsb.org/structure/8APB] (rotational state 1b), 8APC [https://www.rcsb.org/structure/8APC] (rotational state 1c), 8APD [https://www.rcsb.org/structure/8APD] (rotational state 1d), 8APE [https://www.rcsb.org/structure/8APE] (rotational state 1e), 8APF [https://www.rcsb.org/structure/8APF] (rotational state 2a), 8APG [https://www.rcsb.org/structure/8APG] (rotational state 2b), 8APH [https://www.rcsb.org/structure/8APH] (rotational state 2c), 8APJ [https://www.rcsb.org/structure/8APJ] (rotational state 2d), 8APK [https://www.rcsb.org/structure/8APK] (rotational state 3). The local resolution filtered cryo-EM maps, half maps, masks and FSC-curves have been deposited in the Electron Microscopy Data Bank under accession codes: EMD-15560 [https://www.ebi.ac.uk/emdb/EMD-15560] (membrane-region), EMD-15561 [https://www.ebi.ac.uk/emdb/EMD-15561] (peripheral stalk), EMD-15562 [https://www.ebi.ac.uk/emdb/EMD-15562] (rotor), EMD-15559 [https://www.ebi.ac.uk/emdb/EMD-15559] (F1Fo dimer), EMD-15563 [https://www.ebi.ac.uk/emdb/EMD-15563] (rotational state 1a), EMD-15564 [https://www.ebi.ac.uk/emdb/EMD-15564] (rotational state 1b), EMD-15565 [https://www.ebi.ac.uk/emdb/EMD-15565] (rotational state 1c), EMD-15566 [https://www.ebi.ac.uk/emdb/EMD-15566] (rotational state 1d), EMD-15567 [https://www.ebi.ac.uk/emdb/EMD-15567] (rotational state 1e), EMD-15568 [https://www.ebi.ac.uk/emdb/EMD-15568] (rotational state 2a), EMD-15570 [https://www.ebi.ac.uk/emdb/EMD-15570] (rotational state 2b), EMD-15571 [https://www.ebi.ac.uk/emdb/EMD-15571] (rotational state 2c), EMD-15572 [https://www.ebi.ac.uk/emdb/EMD-15572] (rotational state 2d), EMD-15573 [https://www.ebi.ac.uk/emdb/EMD-15573] (rotational state 3). The TEM micrographs of thin cell sections are available from the authors upon request. All other data are available in the article, Supplementary Information or the Source Data file. Source data are provided with this paper.
The atomic coordinates that were used in this study: 6TDU [https://www.rcsb.org/structure/6TDU] (E. gracilis mitochondrial ATP synthase), 6TDV [https://www.rcsb.org/structure/6TDV] (E. gracilis mitochondrial ATP synthase, membrane region), 6B2Z [https://www.rcsb.org/structure/6B2Z] (S. cerevisiae mitochondrial ATP synthase), 6F5D [https://www.rcsb.org/structure/6F5D] (T. brucei F1), 6ZNA [https://www.rcsb.org/structure/6ZNA] (S. scrofa mitochondrial ATP synthase)

Field-specific reporting

Please select the one below that is the best fit for your research. If you are not sure, read the appropriate sections before making your selection.

☒ Life sciences ☐ Behavioural & social sciences ☐ Ecological, evolutionary & environmental sciences

For a reference copy of the document with all sections, see nature.com/documents/nr-reporting-summary-flat.pdf

Life sciences study design

All studies must disclose on these points even when the disclosure is negative.

Sample size	A sufficient sample size was chosen based on previous similar studies, and thus a total of 5,199 movies were recorded and analyzed. No statistical analyses has been performed. The number of cryo-EM particles in the single dataset collected was the number of particles available. No predetermined sample size was used for other experiments.
Data exclusions	Particles that were not ATP synthase were excluded in the analysis, since they cannot contribute to reconstruction.
Replication	Similar cryo-EM structures were successfully obtained from three preliminary datasets.
Randomization	Particle images were randomly assigned into half-sets to obtain gold-standard resolution estimates as described in the text.
Blinding	N/A to cryo-EM study; raw micrographs or particle images are not categorical data. Particles are randomly assigned into half-sets for image processing; hence no blinding is applicable.

Reporting for specific materials, systems and methods

We require information from authors about some types of materials, experimental systems and methods used in many studies. Here, indicate whether each material, system or method listed is relevant to your study. If you are not sure if a list item applies to your research, read the appropriate section before selecting a response.

Materials & experimental systems

n/a	Involved in the study	
☐	☒	Antibodies
☐	☒	Eukaryotic cell lines
☒	☐	Palaeontology and archaeology
☒	☐	Animals and other organisms
☒	☐	Human research participants
☒	☐	Clinical data
☒	☐	Dual use research of concern

Methods

n/a	Involved in the study	
☒	☐	ChIP-seq
☒	☐	Flow cytometry
☒	☐	MRI-based neuroimaging

Antibodies

Antibodies used	All primary antibodies are custom-made by Davids Biotechnologie GmbH, Regensburg, Germany. Primary rabbit anti-subunit beta, -p18, -ATPTB1, -subunit-d and primary monoclonal anti-mtHsp70 as referenced in the text.

	Secondary horseradish peroxidase (HRP) conjugated anti-rabbit and anti-mouse antibody (BioRad).
Validation	Primary antibodies have been validated by probing whole cell lysate and/or mitochondrial lysate from cultured Trypanosoma brucei cells. The specificity is verified by loss of signal in lysates from cells with RNAi knocked-down expression of the expected antigen, where available.<br>The full names and catalogue numbers for the two secondary antibodies are: Goat Anti-Rabbit IgG (H+L)-HRP Conjugate #1721019, Goat Anti-Mouse IgG (H+L)-HRP Conjugate #1721011

Eukaryotic cell lines

Policy information about cell lines

Cell line source(s)	Trypanosoma brucei procyclic Lister 427 strain
Authentication	PCR-based test
Mycoplasma contamination	N/A
Commonly misidentified lines<br>(See ICLAC register)	None
