# Supplementary material for: An ancestral interaction module promotes oligomerization in divergent mitochondrial ATP synthases
Source: Nat Commun. 2022 Oct 11;13:5989. doi: 10.1038/s41467-022-33588-z (PMC9553925; doi:10.1038/s41467-022-33588-z)

Supplementary Fig. 9b

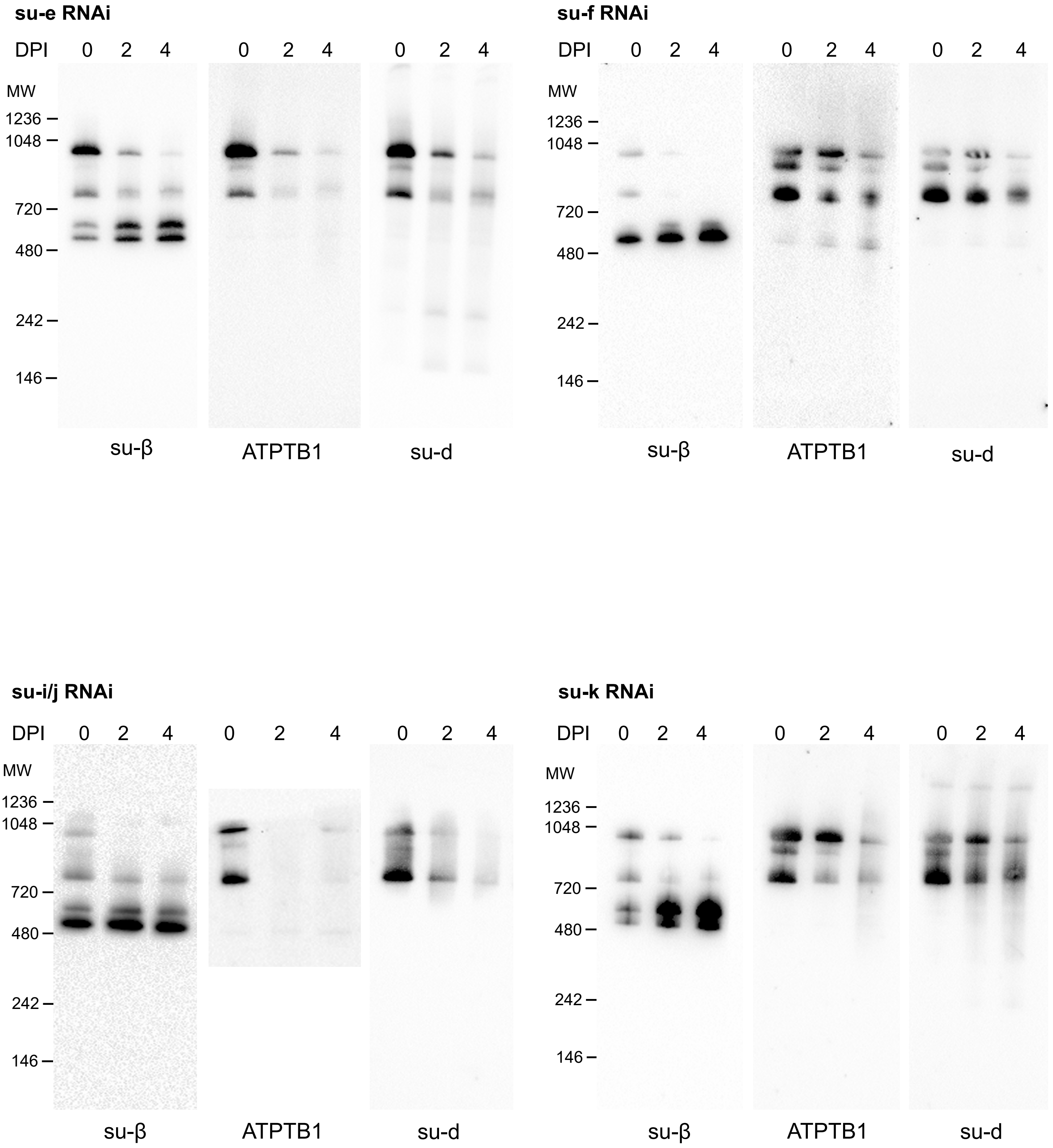

Supplementary Fig. 9b

ATPTB3 RNAi

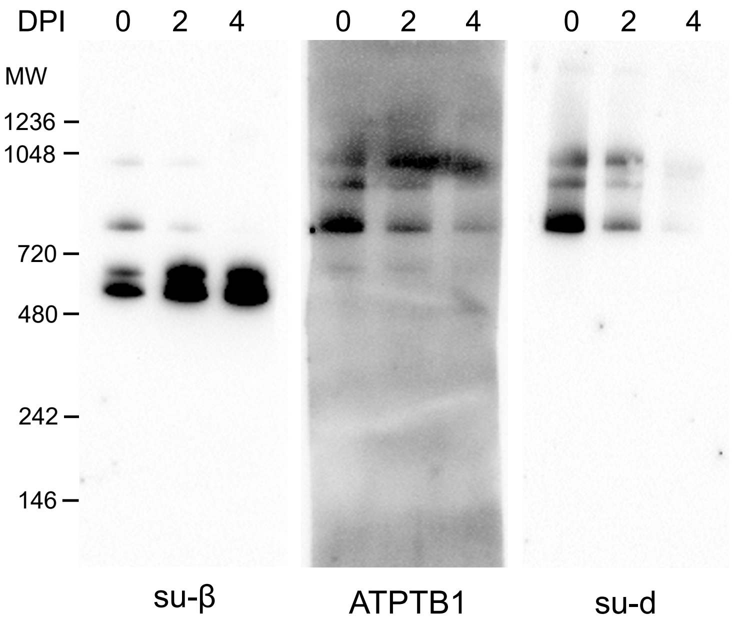

ATPTB6 RNAi

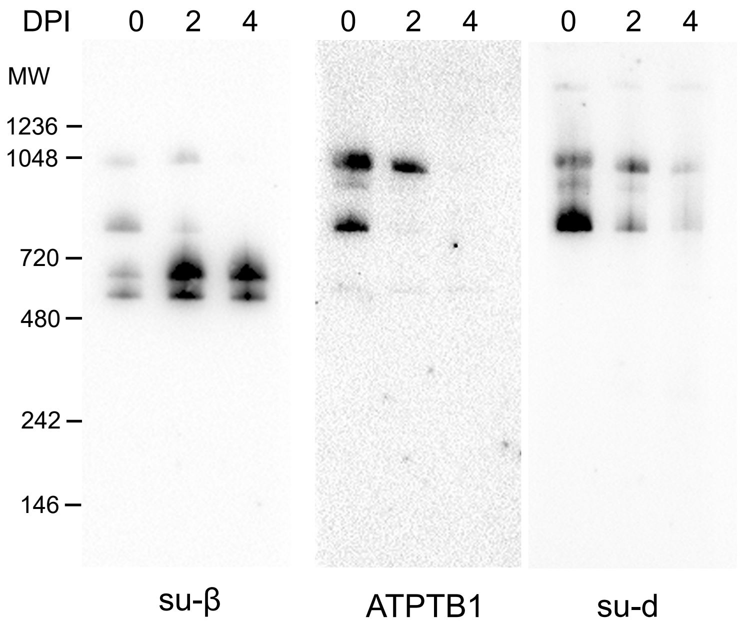

ATPTB12 RNAi

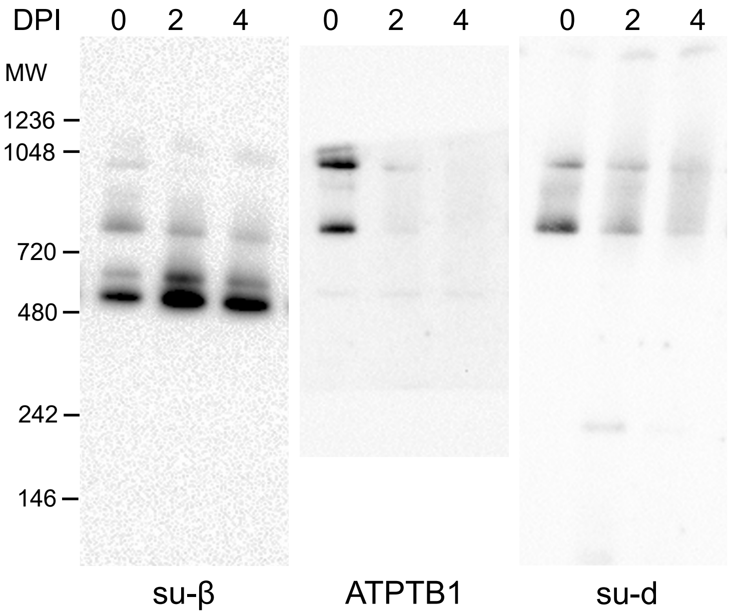

ATPTB14 RNAi

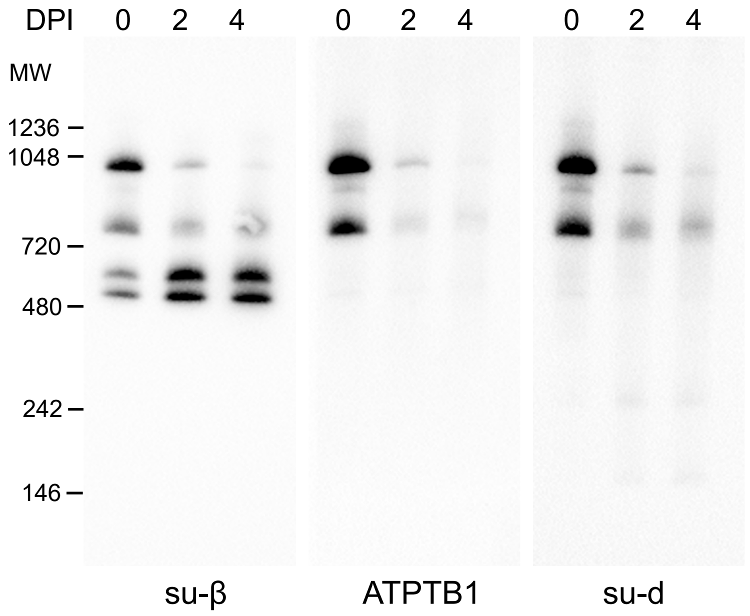

Supplementary Fig. 9b

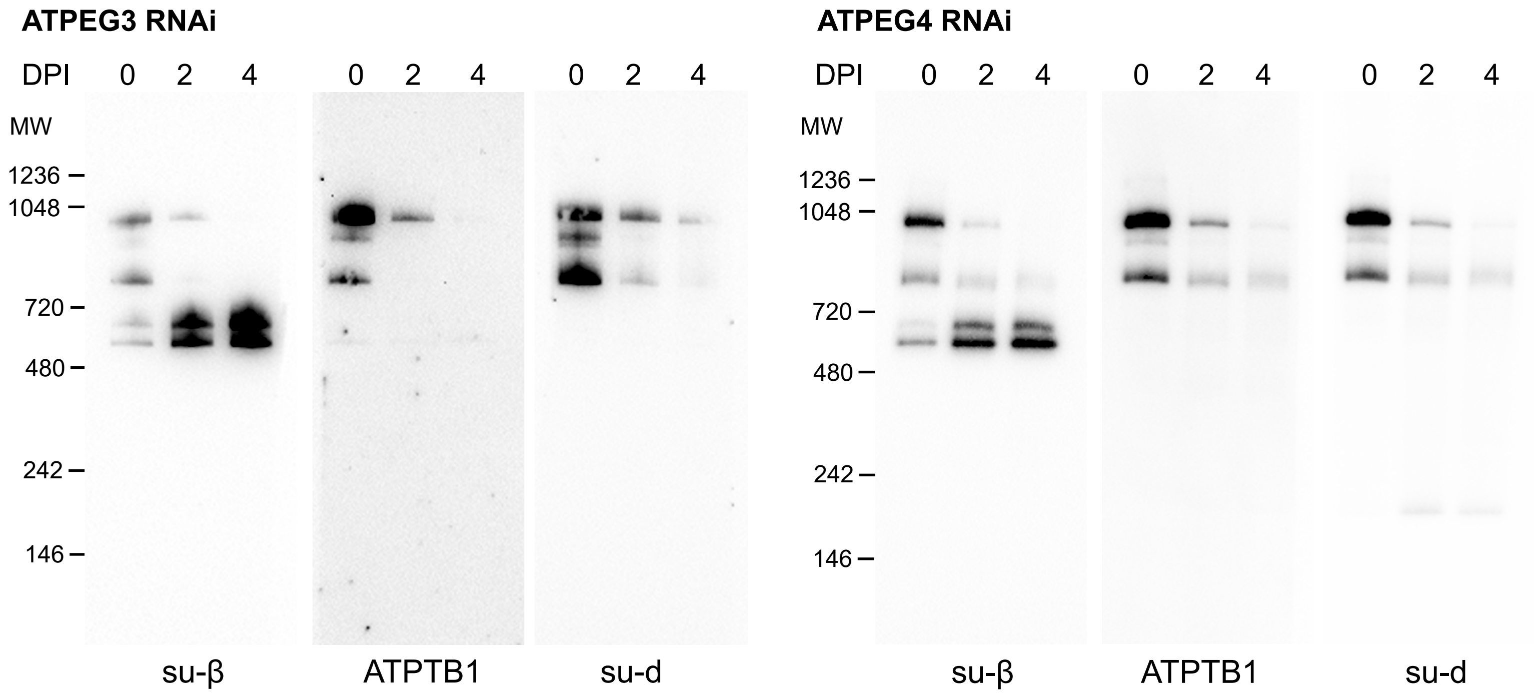

Supplementary Fig. 9c

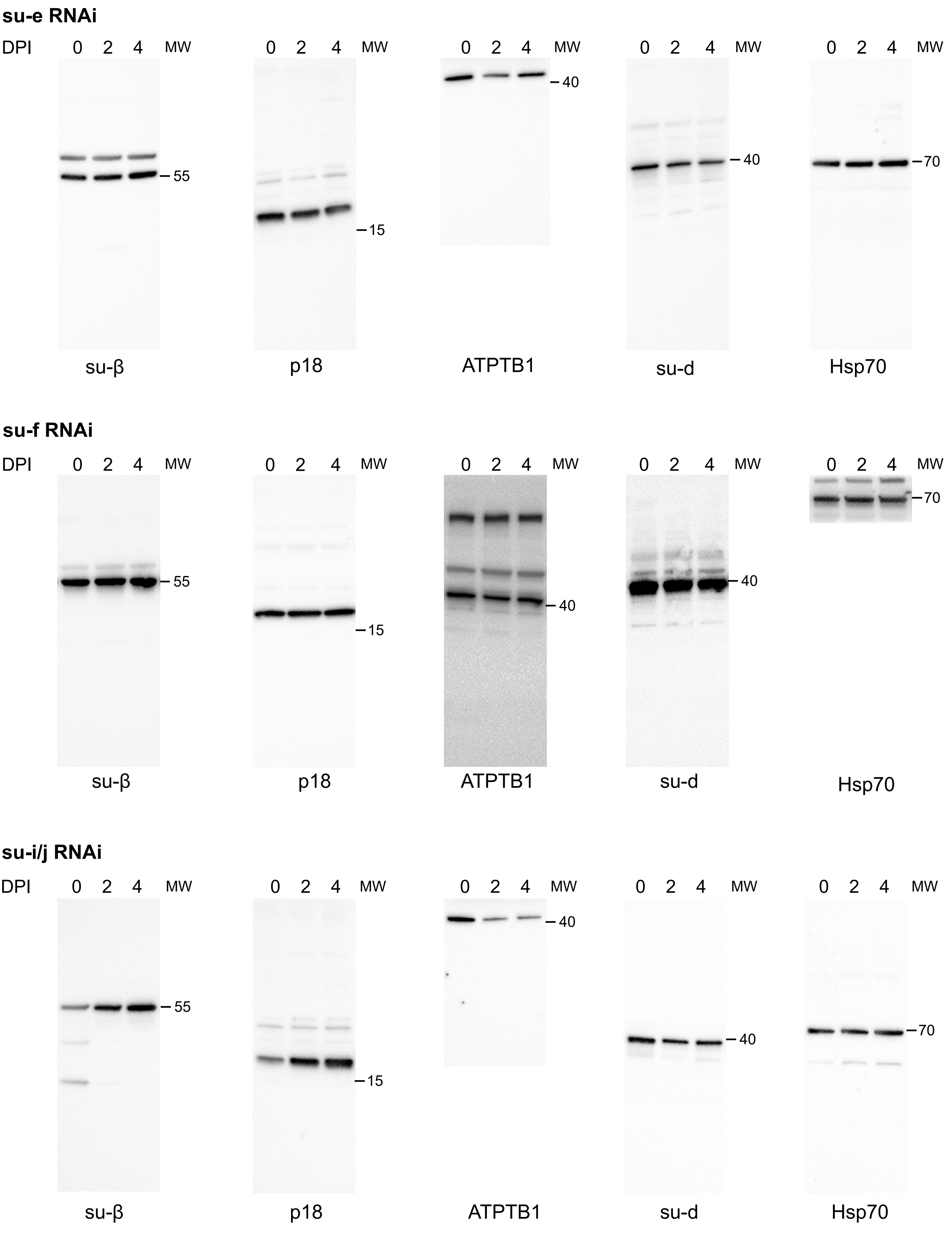

Supplementary Fig. 9c

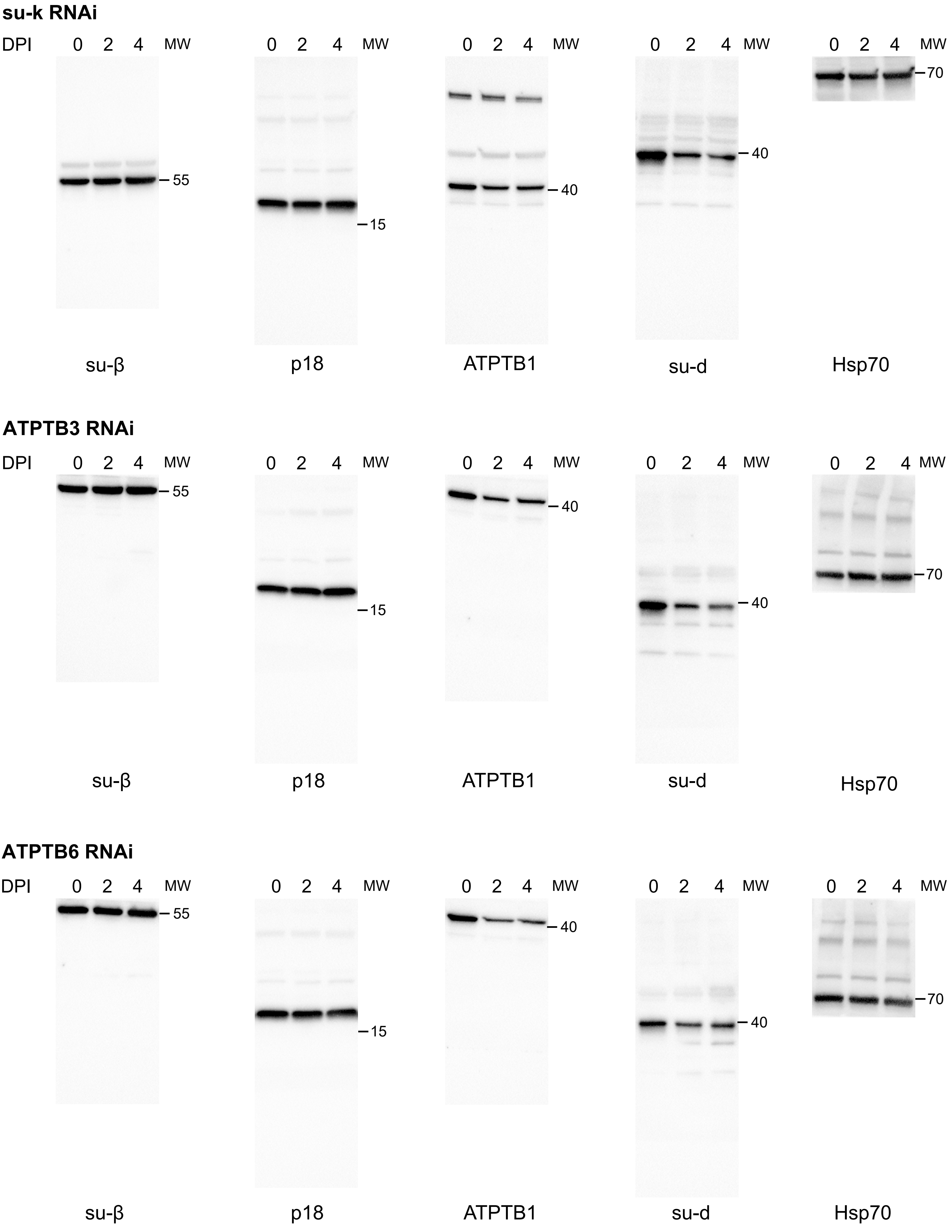

Supplementary Fig. 9c

ATPTB12 RNAi

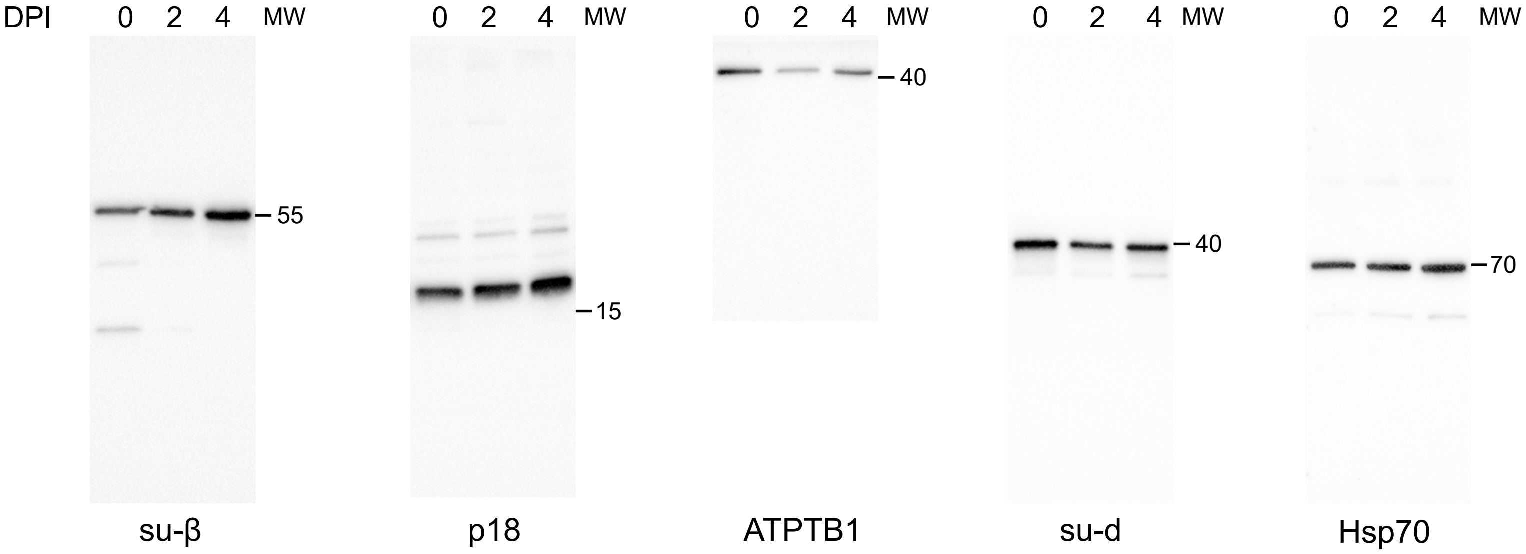

ATPTB14 RNAi

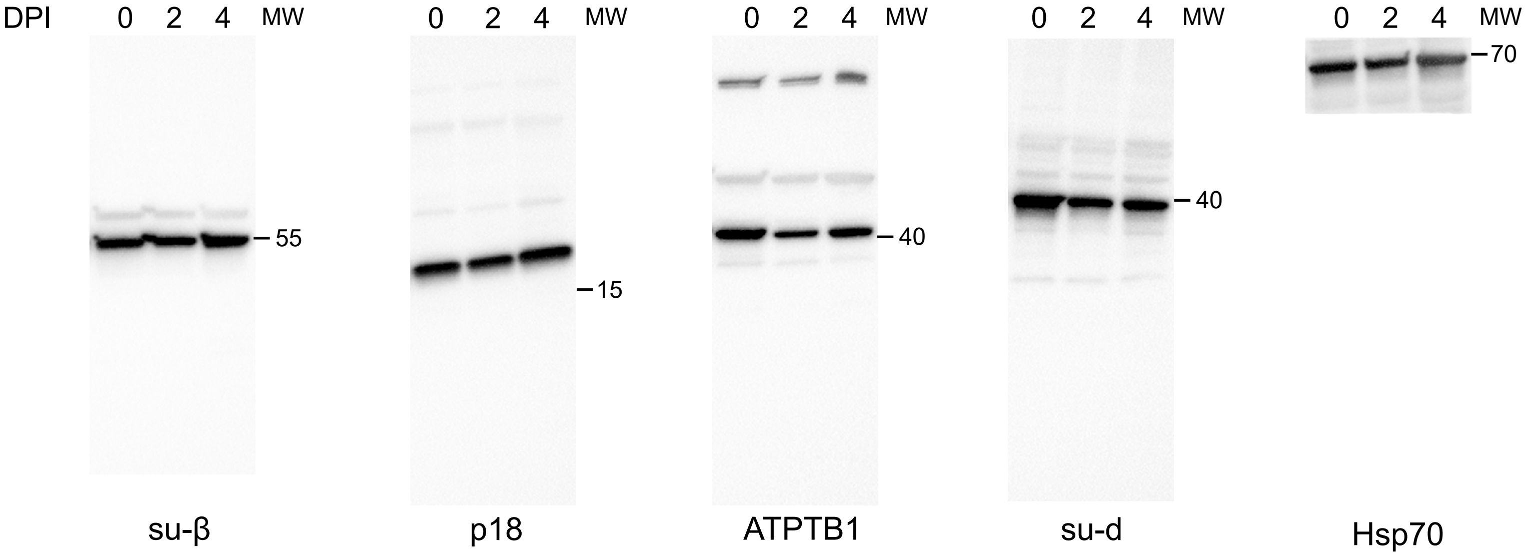

ATPEG3 RNAi

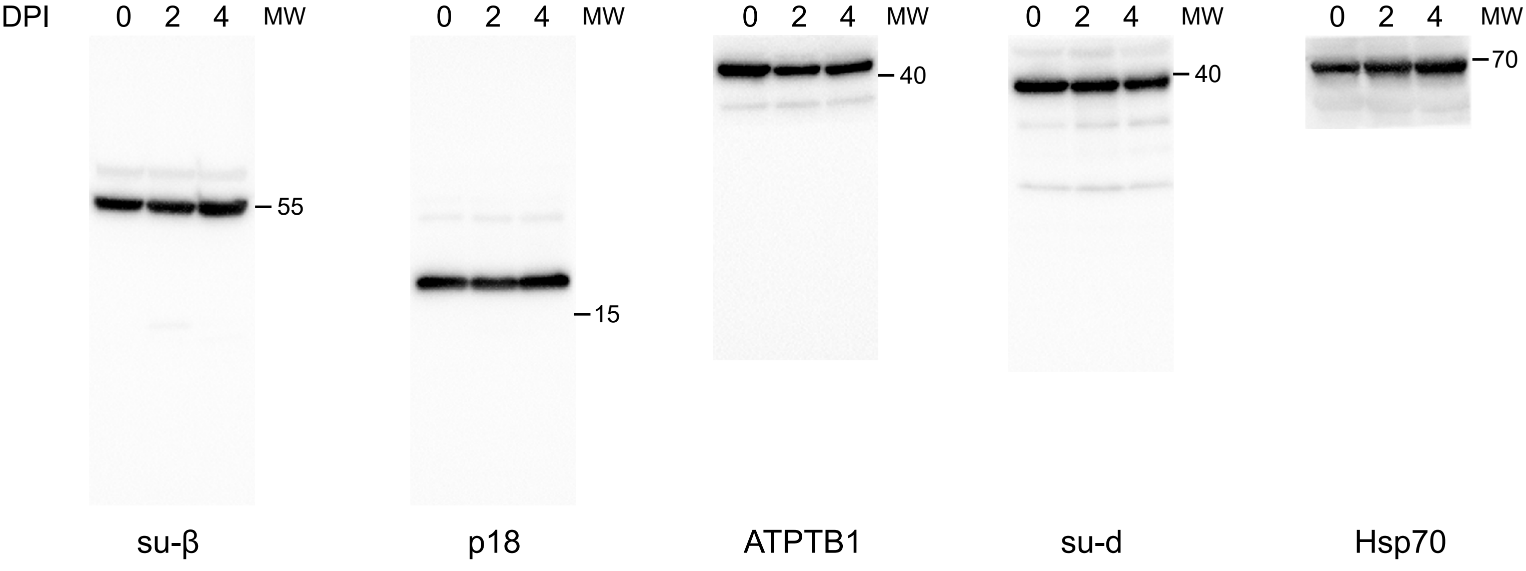

Supplementary Fig. 9c

ATPEG4 RNAi

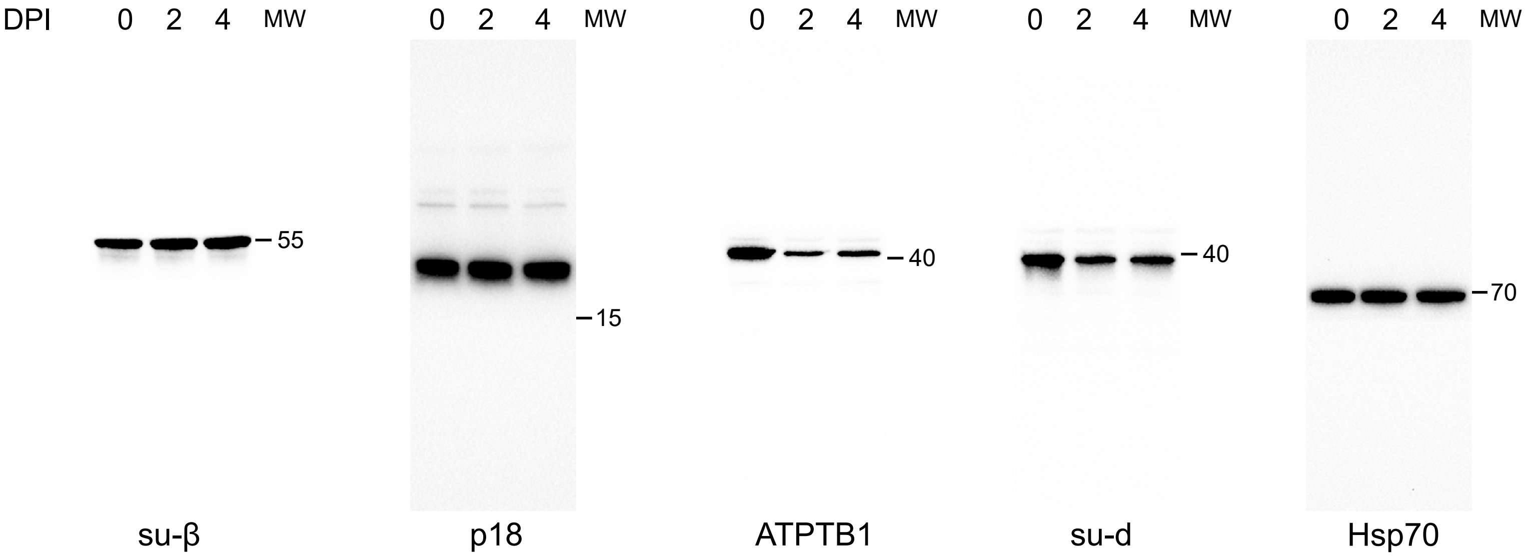

Supplement: Supplementary file 8 — Source Data [file 41467_2022_33588_MOESM8_ESM.zip › Supplementary Fig.9.pdf]
