# Supplementary figures and images for: An ancestral interaction module promotes oligomerization in divergent mitochondrial ATP synthases
Source: Nat Commun. 2022 Oct 11;13:5989. doi: 10.1038/s41467-022-33588-z (PMC9553925; doi:10.1038/s41467-022-33588-z)

Fig. 6b

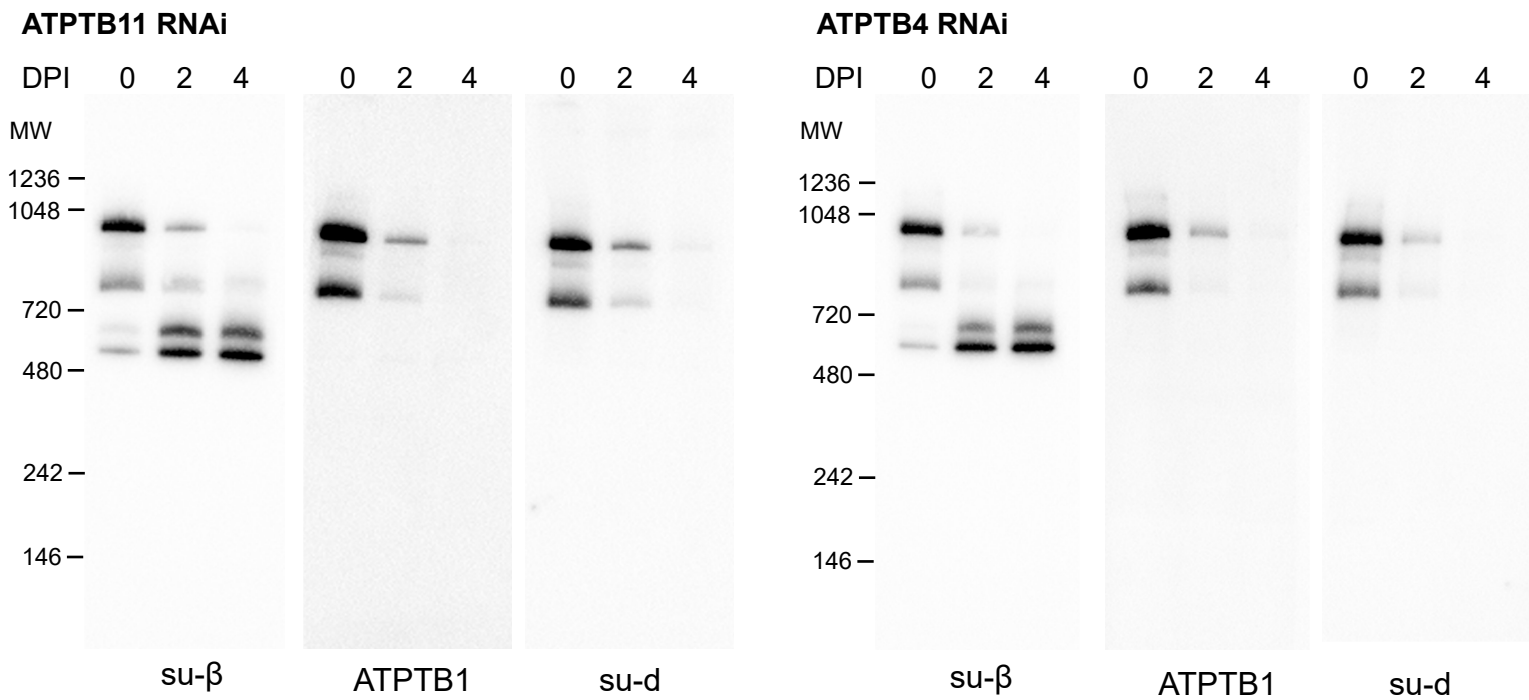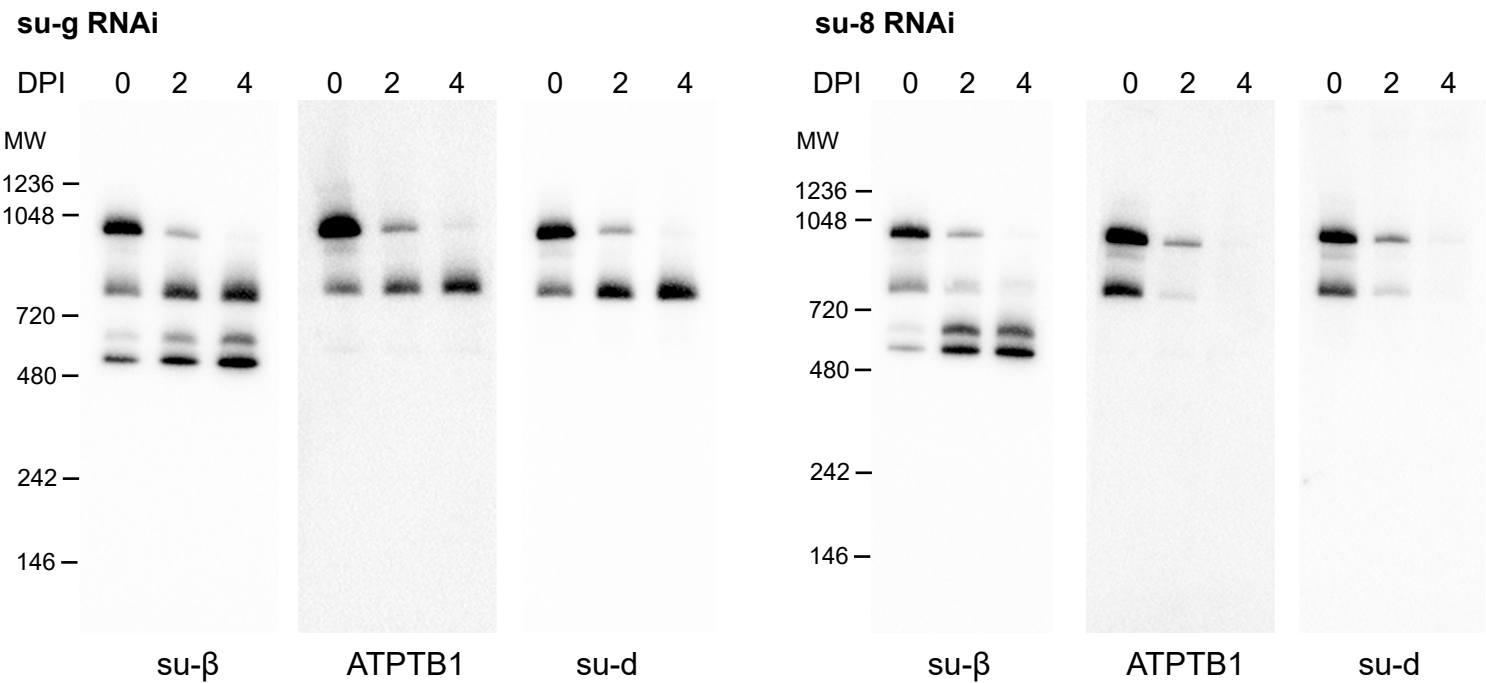

Fig. 6c

ATPTB11 RNAi

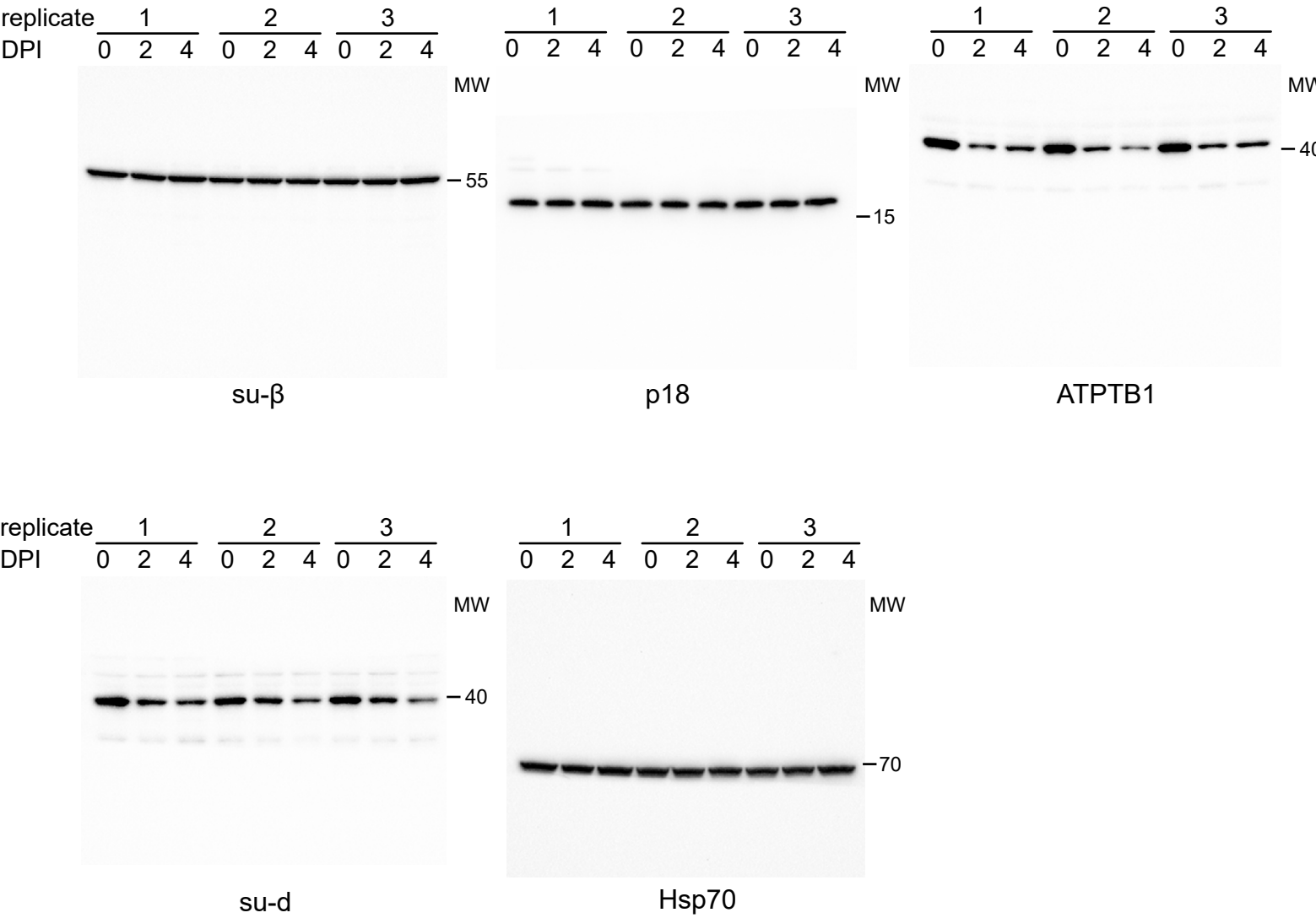

Fig. 6c

ATPTB4 RNAi

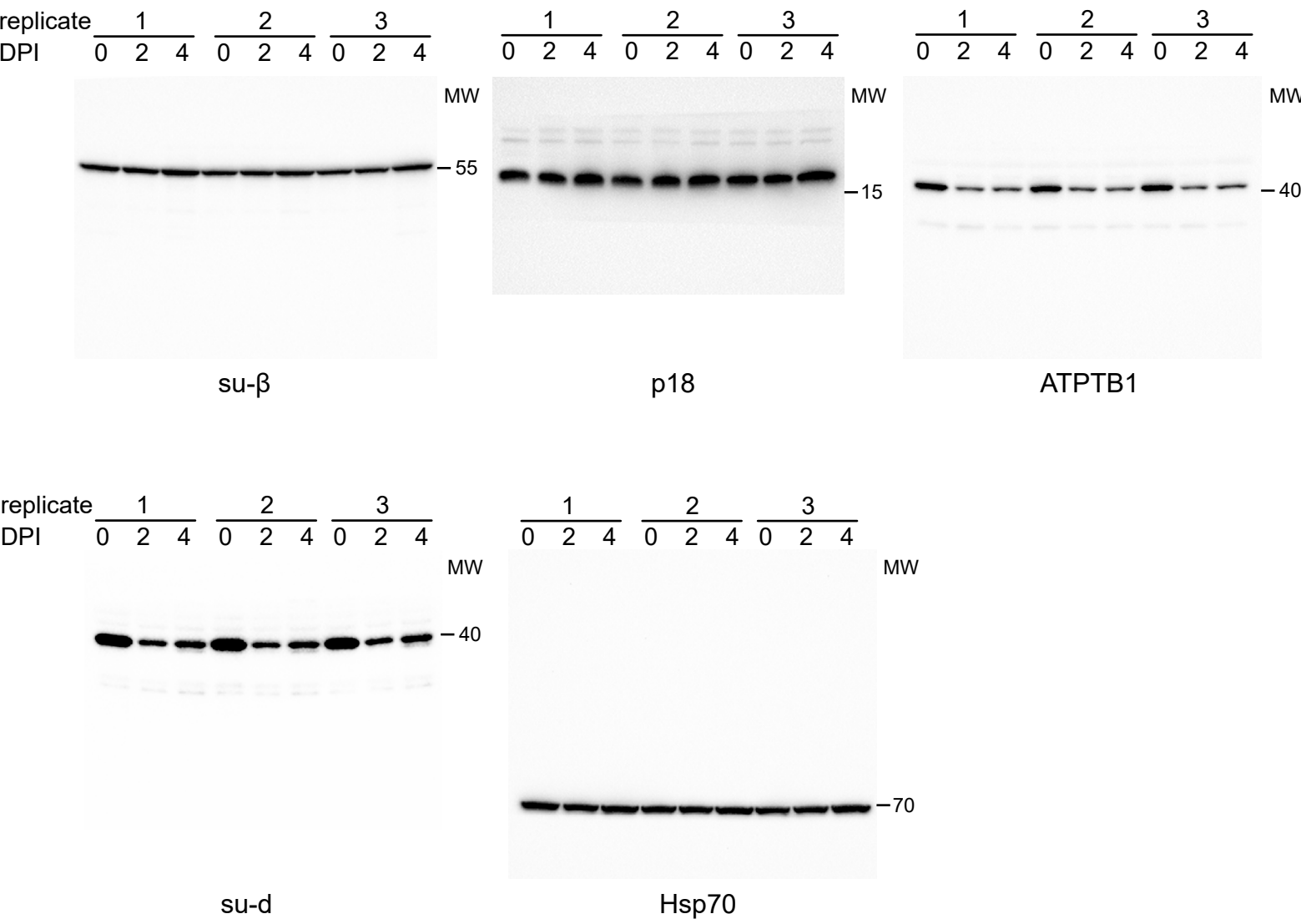

Fig. 6c

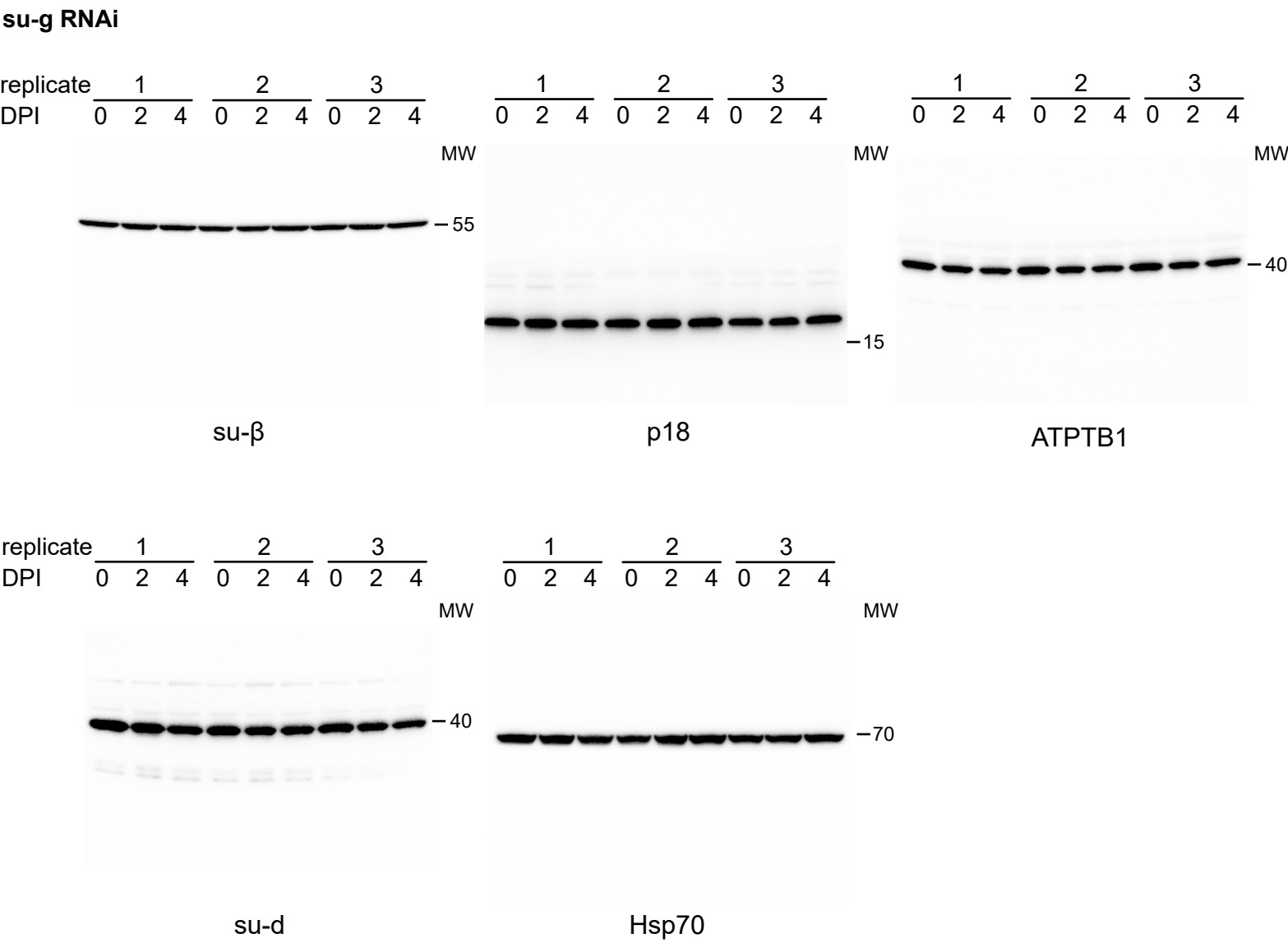

Fig. 6c

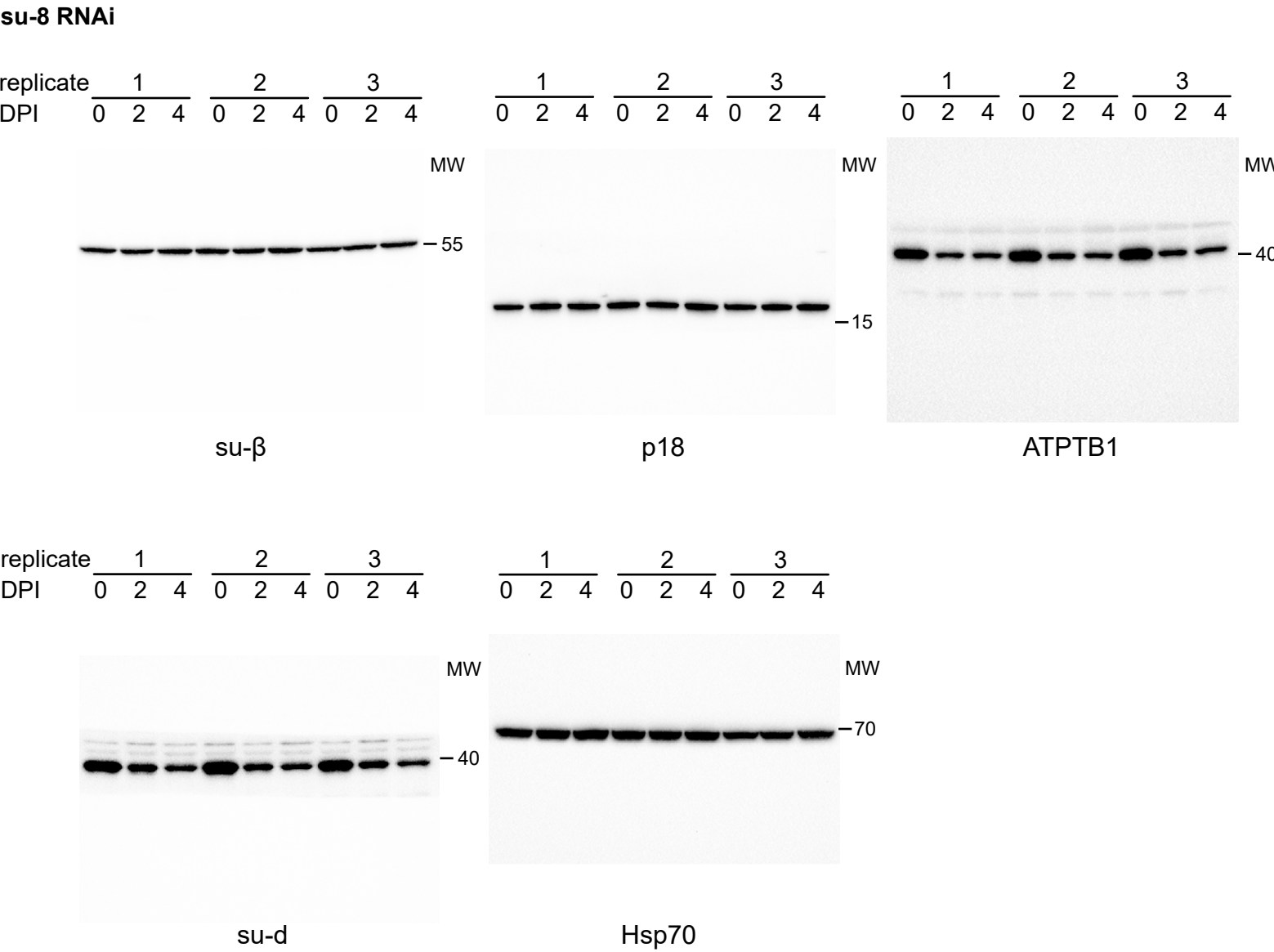

Supplement: Supplementary file 8 — Source Data [file 41467_2022_33588_MOESM8_ESM.zip › Fig.6.pdf]
